# Quantum refinement in real and reciprocal space using the *Phenix* and *ORCA* software

**DOI:** 10.1107/S2052252524008406

**Published:** 2024-09-30

**Authors:** Kristoffer J. M. Lundgren, Octav Caldararu, Esko Oksanen, Ulf Ryde

**Affiliations:** a Department of Computational Chemistry Lund University Chemical Centre, PO Box 124 SE-221 00Lund Sweden; University of Michigan, USA

**Keywords:** X-ray crystallography, neutron crystallography, cryo-EM, quantum refinement, QM/MM, Fe-nitrogenase, V-nitrogenase, Mn superoxide dismutase, particulate methane monooxygenase

## Abstract

X-ray and neutron crystallography, as well as cryogenic electron microscopy (cryo-EM), are the most common methods to obtain atomic structures of biological macromolecules. A feature they all have in common is that, at typical resolutions, the experimental data need to be supplemented by empirical restraints, ensuring that the final structure is chemically reasonable. The restraints are accurate for amino acids and nucleic acids, but often less accurate for substrates, inhibitors, small-molecule ligands and metal sites, for which experimental data are scarce or empirical potentials are harder to formulate. This can be solved using quantum mechanical calculations for a small but interesting part of the structure. Such an approach, called quantum refinement, has been shown to improve structures locally, allow the determination of the protonation and oxidation states of ligands and metals, and discriminate between different interpretations of the structure. Here, we present a new implementation of quantum refinement interfacing the widely used structure-refinement software *Phenix* and the freely available quantum mechanical software *ORCA*. Through application to manganese superoxide dismutase and V- and Fe-nitro­genase, we show that the approach works effectively for X-ray and neutron crystal structures, that old results can be reproduced and structural discrimination can be performed. We discuss how the weight factor between the experimental data and the empirical restraints should be selected and how quantum mechanical quality measures such as strain energies should be calculated. We also present an application of quantum refinement to cryo-EM data for particulate methane monooxygenase and show that this may be the method of choice for metal sites in such structures because no accurate empirical restraints are currently available for metals.

## Abstract 2

We present a new implementation of quantum refinement interfacing the widely used *Phenix* and *ORCA* software. We show applications on a neutron structure of Mn superoxide dismutase, X-ray structures of V- and Fe-nitrogenase and a cryo-EM structure of particulate methane monooxygenase.

## 1. Introduction

Structural information is key to all studies of biomacro­mol­ecular function and mechanisms. Traditionally, the vast majority of atomic-level structural information has come from X-ray crystallography (Rhodes, 2006 ▸). However, cryogenic electron microscopy (cryo-EM) has recently become an important complement (Henderson, 2015 ▸; Nogales, 2016 ▸; Orlov *et al.*, 2017 ▸). It has the advantage that no crystals are needed, but the resolution has been lower, although recent technical advancements have led to great improvements. Neutron crystallography is also an important complement to both X-ray crystallography and cryo-EM because it provides direct information about the positions of hydrogen atoms (Blakeley, 2009 ▸; Schröder & Meilleur, 2021 ▸).

The three methods have many aspects in common and they partly use the same software for structure refinement (Afonine *et al.*, 2010 ▸, 2018 ▸; Liebschner, Afonine *et al.*, 2023 ▸). In particular, all give rise to a density map, into which an atomic model of the macromolecule is built. This model is optimized by minimizing the difference between the experimental data and the corresponding data calculated from the model, a procedure called refinement (Brünger & Rice, 1997 ▸; Kleywegt & Jones, 1997 ▸; Urzhumtsev & Lunin, 2019 ▸).

However, there are also differences among the methods. With cryo-EM, the primary product is the electrostatic-potential scattering maps in real space (Afonine *et al.*, 2018 ▸). Consequently, the refinement is most naturally performed in real space. On the other hand, the primary product of the two crystallographic methods is structure factors in reciprocal (Fourier) space. Unfortunately, the phase information is not available and needs to be obtained from separate experiments or structures of related macromolecules. The detailed maps are built using phase information from the current model and they will therefore change if the model changes (the maps are biased by the model). The maps are electron or nuclear scattering length density maps, for X-ray and neutron crystallography, respectively. Consequently, the refinement is performed in reciprocal space against the structure factor amplitudes.

Another common aspect of the three methods is that there are typically not enough experimental data to specify the exact position of all atoms in the structure. Therefore, the experimental data are normally supplemented by prior knowledge in the form of empirical restraints. These can be experimental information about typical bond lengths, bond angles, torsion angles, chirality and planarity, as well as non-bonded interactions (Engh & Huber, 1991 ▸, 2012 ▸; Moriarty *et al.*, 2016 ▸). In terms of computational chemistry, this is a molecular mechanics (MM) force field. For low-resolution cryo-EM structures, additional information is included, such as secondary-structure (Headd *et al.*, 2012 ▸) and rotamer-specific restraints, as well as information about internal symmetry.

Consequently, the target function that is optimized in the refinement contains two terms: 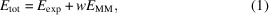
where *E*_exp_ is the experimental target function, describing how well the current model reproduces the experimental raw data, and *E*_MM_ are the empirical restraints. The weight factor, *w*, determines the relative importance of the two terms. Various procedures are implemented in refinement software to select a proper value of *w*, either to give terms of a comparable magnitude or to optimize some quality criterion (Jack & Levitt, 1978 ▸; Brünger *et al.*, 1989 ▸; Brünger & Rice, 1997 ▸; van Zundert *et al.*, 2021 ▸; Afonine *et al.*, 2011 ▸).

Refinement with empirical restraints works quite well if *E*_MM_ is accurate. This is typically the case for amino acids and nucleic acids, for which a large amount of experimental and computational-chemistry information is available. However, for non-standard parts of the macromolecules, such as substrates, cofactors, ligands and inhibitors, much less information is available. Even worse, for metal sites and reaction intermediates with unusual chemical bonding, it is hard to build up reliable MM force fields (Hu & Ryde, 2011 ▸) and therefore the accuracy of the *E*_MM_ term is low in those cases. Therefore, the accuracy of the resulting structure will be different in different parts of the final structure and non-standard molecules are unfortunately found especially in the active site of the macromolecules, *i.e.* the accuracy is often lowest in the biologically most interesting parts of the structures.

In 2002, we suggested how this problem can be solved by replacing *E*_MM_ with more accurate quantum-mechanical (QM) calculations for a small but interesting part of the structure (called subsystem 1 in the following) (Ryde *et al.*, 2002 ▸). This is done in the same way as with combined QM and MM (QM/MM) calculations (Senn & Thiel, 2009 ▸; Ryde, 2016 ▸):
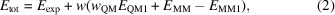
where *E*_QM1_ is the QM energy of subsystem 1, *E*_MM1_ is the MM energy of the same subsystem and *w*_QM_ is another empirical scaling factor, which is needed because the empirical restraints are of statistical nature, whereas *E*_QM1_ is a physical energy. This approach is called quantum refinement (Bergmann *et al.*, 2022 ▸). It was originally developed for X-ray crystallography (Ryde *et al.*, 2002 ▸), but it was later extended to neutron crystallography (Caldararu *et al.*, 2019 ▸), as well as to NMR structure refinement (Hsiao *et al.*, 2005 ▸) and extended X-ray absorption fine-structure (EXAFS) measurements (Hsiao *et al.*, 2006 ▸). We have shown that with quantum refinement we can locally improve crystal structures (Ryde & Nilsson, 2003 ▸), determine protonation states of active-site residues (Nilsson & Ryde, 2004 ▸; Cao *et al.*, 2017 ▸; Bergmann *et al.*, 2021*c* ▸), determine oxidation states of metals (Rulíšek & Ryde, 2006 ▸), detect photoreduction of metal sites (Rulíšek & Ryde, 2006 ▸; Söderhjelm & Ryde, 2006 ▸) and decide which ligands are actually observed in crystal structures (Cao *et al.*, 2020 ▸; Bergmann *et al.*, 2021*b* ▸). Several other groups have implemented similar approaches (Bergmann *et al.*, 2022 ▸; Hsiao *et al.*, 2010 ▸; Fadel *et al.*, 2015 ▸; Yan *et al.*, 2021 ▸). In particular, Merz and coworkers have developed an approach where a linear-scaling semiempirical QM approach is employed for the entire macromolecule (Yu *et al.*, 2005 ▸). This approach (DivCon) has been commercialized by the QuantumBio company and has been employed to improve crystal structures of small drug-like molecules binding to target proteins and decide their proper protonation and tautomeric states (Borbulevych *et al.*, 2016 ▸). Likewise, the Q|R project aims at quantum refinement calculations of complete proteins at the density-functional theory (DFT) level (Zheng, Reimers *et al.*, 2017 ▸; Zheng, Moriarty *et al.*, 2017 ▸; Zheng *et al.*, 2020 ▸; Wang *et al.*, 2020 ▸, 2023 ▸). There are also approaches that use QM calculations to obtain MM parameters for ligands and other sites in crystal structures (Nilsson *et al.*, 2003 ▸; Liebschner, Moriarty *et al.*, 2023 ▸). However, they still use MM restraints during the refinement and therefore do not allow the topology to change, and are less accurate for metal sites and reaction intermediates with unusual chemical bonding.

One issue with our quantum refinement approach, implemented in the *ComQum-X* software (Ryde *et al.*, 2002 ▸), is that it uses outdated crystallographic software, *CNS* (crystallography and NMR system) (Brünger *et al.*, 1998 ▸, 2007 ▸), and commercial software for QM, *Turbomole* (Furche *et al.*, 2014 ▸). Moreover, CNS does not have any support for cryo-EM structures. The DivCon approach has been implemented with the more modern *phenix.refine* software (Adams *et al.*, 2010 ▸; Borbulevych *et al.*, 2014 ▸; Liebschner *et al.*, 2019 ▸), but it is commercial. A Q|R implementation for cryo-EM has been presented (Wang *et al.*, 2020 ▸), but it is computationally very expensive.

In this study, we present a new implementation of quantum refinement, called *QRef*, combining *Phenix* (Liebschner *et al.*, 2019 ▸) and the free QM software *ORCA* (Neese *et al.*, 2020 ▸). The implementation is general and allows for both real-space and reciprocal-space refinement. We describe the implementation and illustrate the capabilities and performance by four simple applications for X-ray crystallography, neutron crystallography and cryo-EM.

## 2. Methods

### 2.1. Implementation

The implementation consists of a preparatory Python script, *qref_prep.py*, as well as a separate Python module, *QRef* (not included with *cctbx* or *Phenix*), which needs to be manually applied to a local *Phenix* installation, following the instructions found at https://github.com/krlun/QRef. More specifically, the constructor of the energies class defined in *cctbx_project/cctbx/geometry_restraints/energies.py* [that normally handles the geometrical restraints in *cctbx* (Grosse-Kunstleve *et al.*, 2002 ▸)] is modified so that the *QRef* module is loaded and is run whenever a request to calculate the gradients from the restraints is made. The *QRef* code then interfaces between *Phenix* and *ORCA* utilizing a subtractive QM/MM scheme for the restraints as described by equation (2). The *w*_QM_ scaling factor was determined by a simple comparison of the numerical values of the bond force constants for the *Phenix* restraints and standard energy-based MM force fields, such as the AMBER ff14SB force field (Maier *et al.*, 2015 ▸). We found that *w*_QM_ = 7.5 mol kcal^−1^ (AMBER uses energy units of kcal mol^−1^) was appropriate and we use this value throughout this study.

In a biological macromolecule, there are typically covalent bonds between the QM and MM subsystems. Since QM calculations require filled valences, the QM region needs to be truncated (capped) in a proper way. This can either be done by adding some atoms or using specialized orbitals (Senn & Thiel, 2009 ▸). We have used the hydrogen link-atom approach (Ryde, 1996 ▸; Reuter *et al.*, 2000 ▸), in which the QM region is truncated by adding a hydrogen atom for each QM–MM bond, called a hydrogen link atom (H_L_), which typically replaces a carbon atom in the MM region, called a carbon link atom (C_L_). We followed standard procedures for the treatment of C_L_ and H_L_ atoms (Maseras & Morokuma, 1995 ▸; Ryde, 1996 ▸); details are given in the supporting information. This is performed automatically by *QRef*.

Alternative conformations outside the QM system are treated by standard procedures in *Phenix*. Alternative conformations inside the QM system require separate QM calculations for each conformation (Cao & Ryde, 2020 ▸). The current implementation allows for multiple QM regions and therefore supports alternative conformations. If a neutron structure has a mixture of H and D atoms within the QM system, they need to be treated as alternative conformations, one with H and one with D, although the QM energy and gradient calculations are indifferent to isotopes.

One special case is not yet implemented: atoms in the QM system on special positions. Note that *Phenix* involves numerous options. The current version of *QRef* has mainly been tested with default options for both reciprocal and real-space refinement. Non-default options should be used with care. For example, we expect that quantum refinement would work with simulated annealing, but it would be very time-consuming. We discourage the use of user-defined bonds within the QM system, because the QM calculations should provide a better description. Moreover, non-crystallographic symmetry constraints involving atoms in the QM system are unlikely to function correctly. Likewise, water picking should be avoided during quantum refinement because the numbering of the QM atoms may change if some atoms are added or deleted. Since quantum refinement is run at the end of a normal refinement and keeps everything fixed except the QM system, non-default options should not be needed.

### 2.2. Usage

To run quantum refinement in *Phenix* using *QRef*, one first needs to select the QM region, *i.e.* the site of interest (there may be several disjoint or overlapping QM subsystems). The selection of the QM system should follow best practices of QM/MM calculations (*e.g.* not cleave conjugated systems), include all metal ligands and preferably cleave only C—C bonds (Senn & Thiel, 2009 ▸; Ryde, 2016 ▸; Chung *et al.*, 2015 ▸). In addition, the QM system needs to be fully protonated, even if the starting structure does not contain any H atoms. The H atoms should be added to the starting PDB file. Each QM region is defined by the user in a text file, listing the serial numbers of atoms from the PDB file that are inside the QM region. Additionally, the user must create a second list within the same text file(s), specifying which atoms in the QM system are chemically bonded to atoms in the surroundings. It is crucial that the atoms in the input model maintain the same order used internally by *Phenix*. This can be achieved by first sorting the input model using the *Phenix* command *iotbx.pdb.sort_atom*.

Using these files, *qref_prep.py* produces a JSON settings file (qref.dat) containing among other things the parameters needed to determine the position of the H_L_ atoms [*g*_bond_ in equation (S2) of the supporting information], which are obtained using a database of optimum QM distances and the force field parameters used by *Phenix*. The script also produces two PDB files for each QM subsystem, one with C_L_ atoms, serving as the input for the calculation of the *E*_MM1_ term, the other with H_L_ atoms, serving as the input for the calculation of the *E*_QM1_ term. These files can also be used to check that the selection of the QM region is correct. Moreover, it produces suggested selection strings for the refinement jobs to allow only coordinates close to the QM region to move during the refinement. For reciprocal-space refinement (through *phenix.refine*), this is achieved by defining which atoms are allowed to move during the refinement. For real-space refinement (through *phenix.real_space_refine*), it is instead necessary to use self-reference restraints with a high weight. The calculations in this study used a weight of 10000, which was found to give essentially fixed atoms outside the QM region, without affecting the convergence of the refinement of the QM region.

QM settings for *ORCA* are provided by the user through text files named qm_*i*.inp, where *i* indicates to which QM region the input file belongs (for example, qm_1.inp contains the *ORCA* settings for the first, and possibly only, QM region), placed in the same folder as the other files of the refinement job.

**Figure 1:**
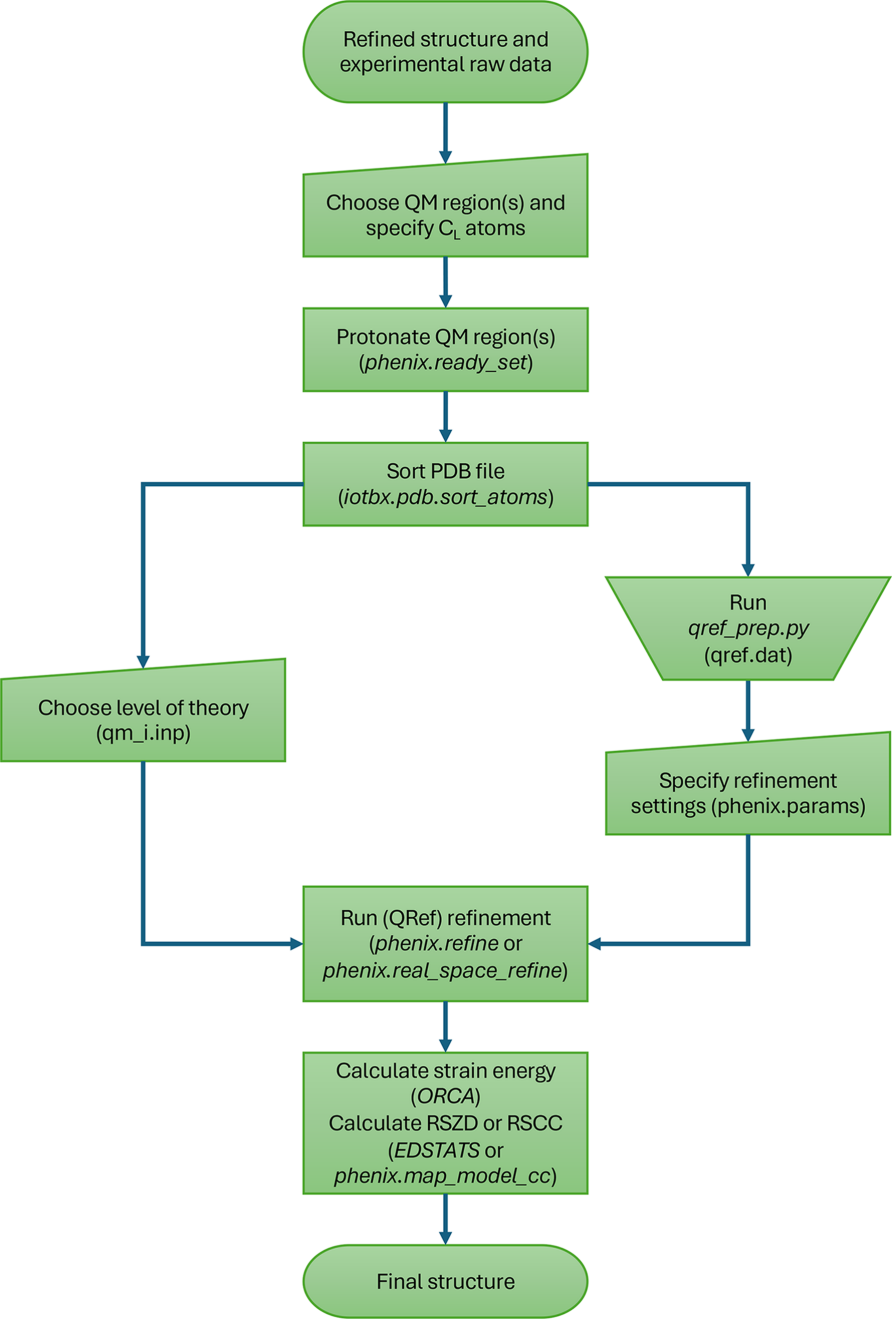
Flow scheme illustrating the *QRef* procedure.

After these setup steps, the quantum refinement is run by executing either *phenix.refine* (Afonine *et al.*, 2012 ▸) or *phenix.real_space_refine* (Afonine *et al.*, 2018 ▸). A flow scheme of the *QRef* procedure is shown in Fig. 1 ▸.

*QRef* has been released under a BSD-3 licence and the source code, supporting scripts, installation and usage instructions, examples, and templates can be found at https://github.com/krlun/QRef. The current version of *QRef* has been verified to work with *Phenix* (versions 1.20.1–4487, 1.21–5207, 1.21.1–5286 and 1.21.2-5419) and *ORCA* (versions 5.0.4 and 6.0.0). We intend to update *QRef* when new versions of *Phenix* are released. More details and a point-by-point description of how to install *QRef* and how to set up quantum refinement calculations are given in the README.md file in the GitHub repository and on the https://signe.teokem.lu.se/ulf/Methods/qref.html page.

### 2.3. Applications

We have applied the new implementation of *QRef* to four protein structures. For all four, the coordinates, occupancies, *B* factors and structure factors were downloaded from the Protein Data Bank (Berman *et al.*, 2000 ▸), together with the space group, unit-cell parameters, resolution limits, *R* factors and the test set used for evaluation of the *R*_free_ factor. Protonation of the QM region was done with *phenix.ready_set* (for Mn superoxide dismutase the deposited model was already deuterated). Restraint files for the non-standard ligands (homocitrate, the P-cluster and the FeV or FeFe clusters in nitro­genase, as well as lipids in particulate methane monooxygenase) were generated using *phenix.elbow* (Moriarty *et al.*, 2009 ▸).

For the three crystal structures, reciprocal-space refinement was performed with *phenix.refine*, involving the first three macrocycles of coordinate refinement, in which only the coordinates of the QM region, as well as the part of the residues outside of the QM region where a covalent bond was cut, were allowed to move, followed by three macrocycles of individual atomic displacement parameter (ADP) refinement, where the ADPs for the whole protein were allowed to change. After that, real-space *Z* scores based on the difference maps (RSZD), real-space *R* factors (RSR) and real-space correlation coefficients (RSCC) were calculated using *EDSTATS* (Tickle, 2012 ▸).

For the cryo-EM structure of particulate methane monooxygenase (pMMO), real-space refinement was performed with *phenix.real_space_refine*, involving five macrocycles of coordinate refinement, in which the protein outside the QM region was kept in place using self-reference restraints with a weight of 10000. After that, RSCC was calculated on a residue-wise basis using *phenix.map_model_cc*.

All QM calculations were performed with the TPSS density-functional theory method (Tao *et al.*, 2003 ▸) and the def2-SV(P) basis set (Schäfer *et al.*, 1992 ▸). We employed the DFT-D4 dispersion correction (Caldeweyher *et al.*, 2019 ▸). Different broken-symmetry states for the nitro­genase models were obtained with the Flipspin approach in *ORCA*.

QM calculations with DFT methods and split-valence basis sets, such as those employed in this study, typically give metal–ligand distances with an accuracy of 0.01–0.06 Å and the errors are even lower for covalent bonds (Ryde & Nilsson, 2003 ▸; Neese, 2006 ▸; Cao *et al.*, 2018 ▸; Benediktsson & Bjornsson, 2022 ▸). This is better than what is obtained for typical medium- and low-resolution protein crystal structures (Fields *et al.*, 1994 ▸) and this is one of the advantages of quantum refinement. *Phenix* refinement depends on a random seed, which affects the *w* weight factor in equation (1) in particular and can give a variation of metal–ligand bond lengths of 0.05–0.1 Å for the present test cases. With a fixed weight or a specific random seed, *QRef* always gives the same results with the same input files. Therefore, we have selected to report distances in ångstroms to two decimal places (energies in kJ mol^−1^ with no decimal), RSZD scores to one decimal place and RSCC values to three decimal places so that so that trends caused by changes in the weight factor can be followed. However, this probably gives a somewhat over-optimistic view of the accuracy of the data.

## 3. Results and discussion

### 3.1. Implementation

We have implemented quantum refinement using a combination of two widely used and freely available (for academic users) software packages: *ORCA* (Neese *et al.*, 2020 ▸) and *Phenix* (Adams *et al.*, 2010 ▸). In our previous implementation of quantum refinement, *ComQum-X* (Ryde *et al.*, 2002 ▸; Bergmann *et al.*, 2022 ▸), we instead used a combination of *Turbomole* (Furche *et al.*, 2014 ▸) and* CNS* (Brünger *et al.*, 1998 ▸). However, *Turbomole* has since then been commercialized, and *CNS* is outdated and little used.

We have implemented quantum refinement as a new module for *Phenix*, which reproduces the full capacity of our original quantum-refinement approach (Bergmann *et al.*, 2022 ▸; Ryde *et al.*, 2002 ▸), but also opens up for all methods and options available in *Phenix*, here illustrated by the extension of quantum refinement to cryo-EM structures. We use *Phenix* as the driver of the structure refinement (in variance to *ComQum-X*, which employed *Turbomole* for the geometry optimization). This makes the quantum refinement as similar as possible to the standard crystallographic refinement. It is our hope that *QRef* will become a standard option in *Phenix*, routinely used by crystallographers.

This implementation requires a few choices in addition to those done in standard refinement. Naturally, the user needs to specify the QM region, *i.e.* the interesting part of the structure that will be treated by QM calculations (Senn & Thiel, 2009 ▸; Ryde, 2016 ▸; Chung *et al.*, 2015 ▸). A typical size is 50–300 atoms with DFT methods [which we have found to be an appropriate level in terms of accuracy and time consumption (Bergmann *et al.*, 2022 ▸)]. Second, the charge and multiplicity of the QM region need to be specified by the user. Third, the QM method and basis set should be specified. Typically, a DFT method and split-valence basis set would be appropriate. In all applications presented in this article, the TPSS-D4 method (Tao *et al.*, 2003 ▸; Caldeweyher *et al.*, 2019 ▸) and the def2-SV(P) basis set were used (Schäfer *et al.*, 1992 ▸).

There are many different variants of QM/MM methods (Senn & Thiel, 2009 ▸; Ryde, 2016 ▸). Equation (2) shows that we are using a subtractive QM/MM approach (Cao & Ryde, 2018 ▸), *i.e.* performing two separate MM calculations, one for the full system (*E*_MM_) and one for the isolated QM region (*E*_MM1_). The latter term simply cancels the MM calculation for the QM region. This is the easiest QM/MM method to implement and there is no need to modify the MM code or to cherry-pick exactly what MM terms to include in the energy function.

When using the subtractive QM/MM scheme, it is essential that all MM terms involving the QM system cancel exactly between *E*_MM_ and *E*_MM1_. This is the case for normal MM terms, such as bonds, angles and dihedrals, especially when using C_L_ atoms. However, we have noted that it might be a problem for some other MM terms. In particular, we have found that the conformational-dependent library (CDL) restraints may sometimes be problematic, because they depend on the backbone dihedral angles and the backbone is often not included in the QM region. Therefore, we recommend that the CDL restraints are turned off when using quantum refinement. Likewise, secondary-structure restraints should be omitted. Metal coordination restraints should work properly if all ligands of the metals are included in the QM system (as they should), but we recommend that they are turned off (because the metal site is better described by the QM calculations). The same applies to Ramachandran plot restraints, residue side-chain rotamer restraints and other non-default restraints. Removing restraints is acceptable because quantum refinement is intended to be run at the end of the refinement, when the global fold and general structure is already settled, and only the detailed structure of a small part of the structure is of central interest. The remainder of the structure is fixed or kept close to the starting structure. Finally, refinement of the hydrogen atoms should be set to ‘individual’ and not ‘riding’ in the *Phenix* parameter file. Since the coordinates outside the QM system are fixed or heavily restrained to the starting coordinates, this does not introduce any risk of overfitting.

### 3.2. Application on a neutron crystal structure

In the following, we will illustrate the new *QRef* implementation by four typical applications, involving neutron and X-ray crystal structures, as well as a cryo-EM structure. We will start with a neutron-crystallography structure and a discussion of the weight factors.

**Figure 2:**
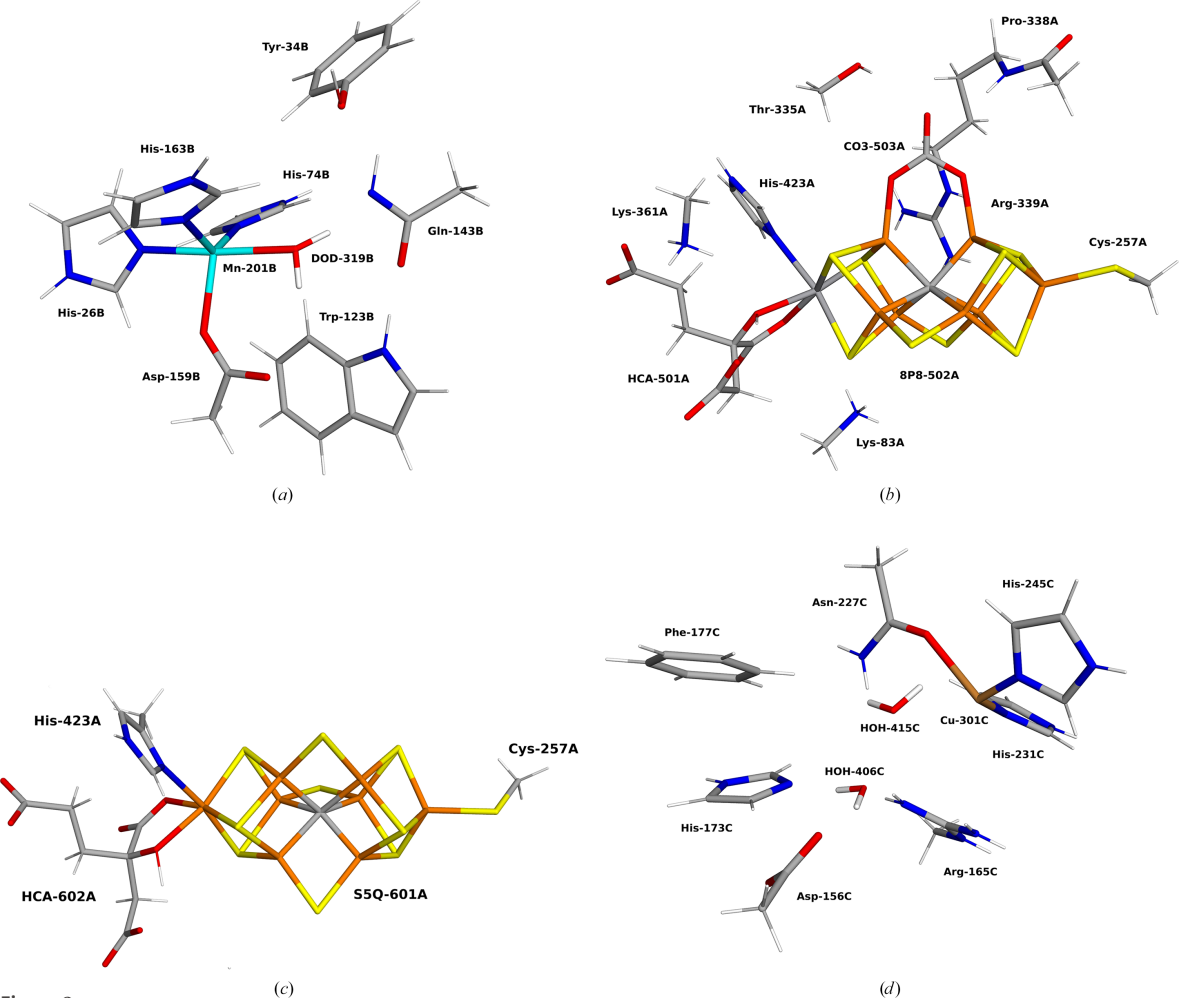
The active sites of the studied systems, showing the QM regions employed: (*a*) MnSOD, (*b*) V-nitro­genase, (*c*) Fe-nitro­genase and (*d*) pMMO.

In 2021, Borgstahl and coworkers published X-ray and neutron structures of Mn superoxide dismutase (MnSOD) in both the reduced (Mn^2+^) and the oxidized (Mn^3+^) states (Azadmanesh *et al.*, 2021 ▸). They showed several conspicuous features, *e.g.* a deprotonated Gln residue, a deprotonated Tyr residue and possibly a doubly deprotonated His residue. The active site consists of the Mn ion, coordinated to three His residues, one Asp residue and a solvent molecule [*cf*. Fig. 2 ▸(*a*)]. Traditionally, it has been assumed that the solvent molecule is water in the reduced state and a hydroxide ion in the oxidized state (Maliekal *et al.*, 2002 ▸; Han *et al.*, 2002 ▸; Miller *et al.*, 2003 ▸; Rulíšek & Ryde, 2006 ▸; Rulíšek *et al.*, 2006 ▸). However, the solvent molecule receives a hydrogen bond from the side chain –NH_2_ group of Gln143, which might be unfavourable for a water molecule. In subunit B of the reduced neutron structure (PDB entry 7kkw), only one proton was seen on Gln143B (the letter after the residue number indicates the chain), *i.e.* Gln143B is deprotonated (–CONH^−^) and receives a hydrogen bond from the Mn-bound water molecule (Azadmanesh *et al.*, 2021 ▸). This is quite unexpected as the p*K*_a_ of water in Mn^2+^(H_2_O)_6_ is appreciably lower than that of acetamide (a simple model of a Gln sidechain), 10.6–10.7 (Yatsimirksii & Vasilev, 1960 ▸; Burgess, 1978 ▸) compared with 15.1 (Haynes, 2016 ▸).

We have studied this reduced neutron structure with *QRef* quantum refinement, using a QM region consisting of [Mn(imidazole)_3_(CH_3_COO)(H_2_O)(phenol)(indole)­(CH_3_CONH)]^−^, as models of His26B, His74B, His163B, Asp159B, Tyr34B, Trp123B and Gln143B [shown in Fig. 2 ▸(*a*)]. Mn^2+^ was modelled in the high-spin state with five unpaired electrons.

As mentioned in the Introduction[Sec sec1], the energy function of *Phenix* reciprocal-space refinement is given by equation (1). In practise, the function is slightly more complicated:
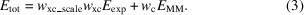
Thus, it contains three weight factors, which determine the relative importance of the crystallographic and geometric-restraint pseudo-energy terms. In principle, two of them are redundant, but they give the user more freedom to vary the importance of the *E*_exp_ and *E*_MM_ terms independently and to turn each of them off without letting a weight go towards infinity, which may lead to convergence problems. The *w*_xc_scale_ term seems to be there for historical reasons and is normally kept at 1/2 (Adams *et al.*, 1997 ▸). In the following, we will only report the product *w*_x_ = *w*_xc_scale_*w*_xc_ and it should be kept in mind that what matters in practice is the quotient *q*_w_ = *w*_x_/*w*_c_.

In the deposited crystal structure (PDB entry 7kkw), the Mn—N_His_ bond lengths to the three His ligands in subunit B are 2.10–2.25 Å, the Asp ligand is monodentate with a Mn—O_Asp_ bond length of 2.16 Å and the solvent molecule is water with an Mn—O_W_ bond length of 2.25 Å, whereas Gln143B is deprotonated [*i.e.* with a –CONH^−^ side-chain group; *cf*. Fig. 2 ▸(*a*)]. The O_W_—H_W_ bond length in this water molecule is 0.97 Å and the length of the H_W_—N_Gln_ hydrogen bond to NE2 of Gln143B is 1.59 Å (the O atom of this water molecule is denoted O_W_, whereas the two H atoms are denoted H_W_ and H_W2_, of which H_W_ forms a hydrogen bond to Gln143B). The average RSZD score for the nine residues in our QM region is 1.3 in the deposited structure, ranging from 0.5 for Tyr34B to 2.5 for His163B (the individual RSZD scores are given in Table S1 of the supporting information). The strain energy (*i.e.* the difference in QM energy of the QM region in the refined structure and a structure obtained by setting *w*_x_ = 0, *i.e.* a QM/MM structure with the *Phenix* MM energy function) is 362 kJ mol^−1^ (with H_L_ atoms added and optimized by QM). If the same structure is freely optimized by QM [TPSS-D4/def2-SV(P)], the Mn—N_His_ bond lengths increase to 2.23–2.35 Å, the Mn–O_Asp_ bond decreases to 2.10 Å and the Mn–O_W_ bond length decreases to 1.99 Å, because H_W_ moves to Gln143B, giving an OH^−^ group (explaining also the longer Mn—N bonds) and a normal neutral Gln143B. The O_W_—H_W_ and H_W_—N_Gln_ distances are 2.67 and 1.07 Å, respectively. This reflects that the expected p*K*_a_ of an amide group [*e.g.* ∼15.1 for acetamide (Haynes, 2016 ▸)] is higher than that of water, 14.0, and the latter can be expected to decrease by several units when coordinating to a metal ion [the p*K*_a_ of Mn^2+^(H_2_O)_6_ is ∼10.6 (Yatsimirksii & Vasilev, 1960 ▸; Burgess, 1978 ▸)].

**Figure 3:**
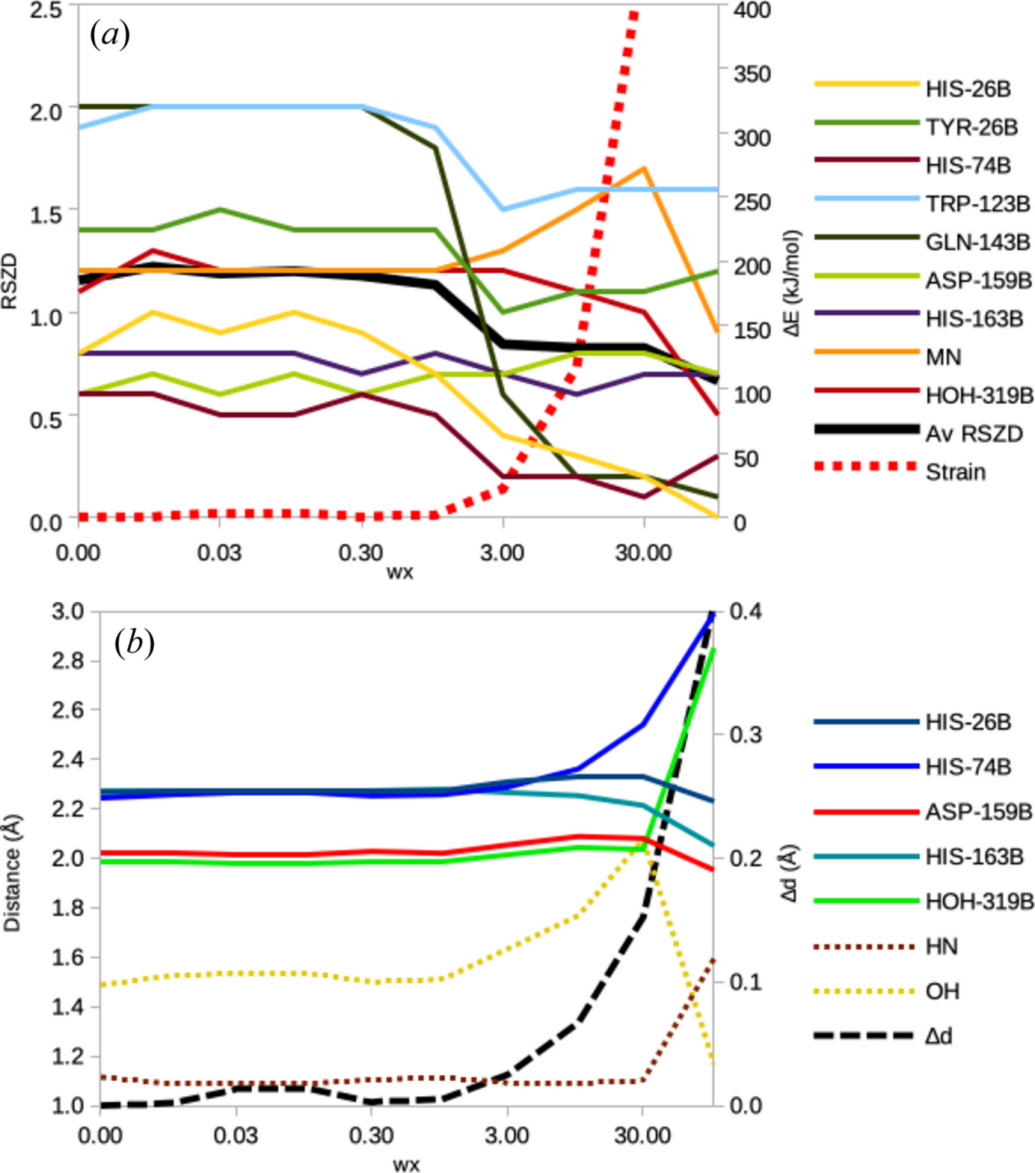
Dependence of (*a*) the RSZD scores (left axis) and the strain energy (right axis, dotted line), as well as (*b*) the bond lengths to Mn (left axis, full lines), distances involving the hydrogen bond between the solvent ligand and Gln143 (left axis and dotted lines) and the deviation of these distances from the QM/MM reference structure (Δ*d*; right axis and dashed line) as a function of the *w*_c_ factor for the neutron structure of reduced MnSOD (7kkw).

We ran several quantum-refinement calculations of MnSOD, varying *w*_x_ but keeping *w*_c_ = 1.0 (and *w*_QM_ = 7.5). The results in Table 1 ▸ and Fig. 3 ▸ show quite a strong dependency on the *w*_x_ weight factor, as expected. When *w*_x_ ≤ 1, the structure is mainly determined by QM. For *w*_x_ = 0.1, the Mn—N_His_ bonds are 2.27 Å and Mn—O_Asp_ = 2.02 Å. Interestingly, the solvent molecule is automatically deprotonated to OH^–^ during the refinement and the proton moves to Gln143B. This shows an advantage of using QM for the restraints: the topology of the system is not fixed, but bonds can break and form following the inherent stability of the various states. Consequently, the Mn—O_W_ bond length decreases to 1.98 Å, O_W_—H_W_ = 1.53 Å and H_W_—N_Gln_ = 1.09 Å. The same applies for *w*_x_ = 0–1 with variations of only 0.01 Å (0.03 Å for Mn—N_His74_). Therefore, the strain energies are small (1–4 kJ mol^−1^) and the average deviation of the seven distances in Table 1 ▸ from the corresponding QM/MM structure (*i.e.* a structure started from the quantum-refined structure, but setting *w*_x_ = 0; Δ*d*_av_) is only 0.000–0.014 Å. The average RSZD score is 1.1–1.2, *i.e.* slightly lower that for the deposited structure. His74B gives the lowest RSZD (0.5–0.6) and Trp123B and Gln143B the highest (1.9–2.0; *cf*. Table S1).

When *w*_x_ ≥ 30 or larger, the structure starts to become chemically unreasonable, with large QM strain energies (>400 kJ mol^−1^) and non-planar ring systems. On the other hand, the average RSZD score decreases from 0.8 to 0.3 when *w*_c_ = 0 (*i.e.* refined with no empirical restraints and no QM). This illustrates the need for empirical (or QM) restraints and that the lowest RSZD scores are obtained for chemically unreasonable structures. At *w*_x_ = 100, H_W_ moves from Gln to the solvent molecule (forming water), with O_W_—H_W_ = 1.17 Å and H_W_—N_Gln_ = 1.59 Å. At *w*_c_ = 0 the structure breaks down completely with the water and two His ligands dissociating from Mn, and H_W2_ pointing towards Mn.

However, for *w*_x_ between 1 and 10, reasonable structures are obtained that reflect a compromise between the crystallographic data and the QM calculations. The strain energies increase from 2 to 117 kJ mol^−1^ and Δ*d*_av_ increases from 0.005 to 0.067 Å. Meanwhile, the average RSZD decreases from 1.1 to 0.8. It is primarily the RSZD scores of Trp123B and Gln143B that decrease. The Mn–ligand distances show variations of 0.03–0.10 Å and the hydrogen-bond distances vary between 1.51 and 1.77 Å (but the proton stays on Gln143B).

Thus, it seems reasonable to select *w*_x_ in this range and the user can make a choice that biases the result either towards crystallography or QM, *e.g.* depending on the accuracy and resolution of the experimental data. In this case, we would suggest a value in the middle of the range, *i.e. w*_x_ = 3, which is similar to the value suggested by *Phenix* in a standard refinement without QM (*w*_x_ = 3.4–5.9 in the last two macrocycles in the refinement procedure). Fig. S1 of the supporting information compares the *mF*_o_ − *DF*_c_ difference maps for the deposited structure and the quantum-refined structure obtained with *w*_x_ = 3. It can be seen that the map of the quantum-refined structure is slightly better, especially between the solvent molecule and Gln143B. However, the difference density is at quite a low level (2.5σ; no differences are observed at 3.0σ for any of the structures). This shows that structures of a comparable quality can be obtained also with a protonated Gln143B. However, the detailed interpretation of the protonation states requires a more involved study including quantum refinement of additional protonation states (in particular with water and a neutral Gln143B). This will be performed in a separate study. The main goal here is to show that the quantum refinement works properly and what variations of the structure and the quality factors can be expected when *w*_x_ is varied.

Finally, we note that the *R*_work_ and *R*_free_ values show little variation among the various structures. *R*_work_ is 22.4–22.7% for all quantum-refined structures, with the lowest values obtained with the highest *w*_x_ (*cf*. Table S1). This is appreciably lower than for the deposited structure (25.1%), but applies to all our *Phenix* calculations, also without QM, indicating slightly different settings for *Phenix*. Likewise, *R*_free_ is 30.4–30.6%, this time with the lowest values obtained for low *w*_x_ values. This is similar to what is reported for the deposited structure (29.9%). The small variation of the *R* values is expected, because they are global measures that are barely affected by changes only in a small part of the protein. Moreover, the variation is similar to the uncertainty in these measures, obtained by refining the structure with different random seeds. Therefore, *R* values will not be discussed in the other applications.

### 3.3. Strain energies

The strain energy is intended to show how close the quantum-refined structure is to the ideal QM structure and can therefore be used to signal if *w*_x_ is too high so that the structure becomes chemically unreasonable, as we saw in the previous section. However, the strain energy depends on the structural interpretation of the crystal structure, including details that are not obvious from the experimental structure, *e.g.* the oxidation state and the location of all protons. Therefore, the strain energies can also be used to decide which of several possible structural interpretations fit the experimental data best: if we use the correct structural interpretation, then the ideal QM structure and the crystal structure should be similar.

However, we then need to decide exactly what we mean by the ‘ideal QM structure’. In the first applications (Ryde *et al.*, 2002 ▸), we simply used the structure of the isolated QM region optimized under vacuum. This is a well-defined structure and works well for small and completely connected QM regions, such as a metal with its first-sphere ligands. However, as the QM region grows bigger, there is a large risk that some groups move significantly during the geometry optimization and may form new interactions (*e.g.* new hydrogen bonds) that are not relevant for the crystal structure. Therefore, in later applications, we instead started to use the QM/MM structure obtained without any crystallographic information as the reference (*i.e.* by setting *w*_x_ = 0). The calculation was typically started from the quantum-refined structure. This is the definition used in the previous section. An alternative is to use QM-optimized structures, but keeping the H_L_ junction atoms fixed.

In this section, we study different choices of reference structures for the strain energies and in particular how they depend on the starting structure (ideally, the reference structure should not depend on which structure the optimization is started from). We used the MnSOD test system and the same ten structures as in the previous section obtained with different values of the *w*_x_ weight (from 0 to 100).

The results in Table 1 ▸, discussed in the previous section, show that the strain energies vary widely with *w*_x_, from 1 to 1 000 000 kJ mol^−1^. These used the structures obtained by carrying out a QM/MM geometry optimization without any crystallographic data as the reference (*i.e.* setting *w*_x_ = 0) and the calculations were started from each quantum-refined structure. Table 2 ▸ shows the individual QM energies of the reference (QM/MM) structures (column *E*_2_). It can be seen that there are some variations in the energies of the reference structures, up to 11 kJ mol^−1^. The calculations started from the quantum-refined structures where *w*_x_ = 0 and 100 give the most and least negative energies, respectively, and the variation is rather random but with a slight trend that the strain energy increases with *w*_x_. Such a large variation was not observed with *ComQum-X*, in which the QM system was optimized by the *Turbomole* software. Using the structure with the lowest QM energy among the ten structures as the reference for all structures seems to be a better choice, giving somewhat more consistent strain energies (column Δ*E*_2_ in Table 2 ▸).

To avoid this problem, we instead tried to obtain reference structures by performing a QM optimization of the QM region under vacuum with *ORCA* and keeping the H_L_ junction atoms fixed. Again, we started all optimizations from the final quantum-refined structure. The results in Table 2 ▸ (column *E*_3_) show that this reduced the variation in the reference energies to 5 kJ mol^−1^ (0.1 kJ mol^−1^ if only structures with *w*_x_ = 0–1 are considered). The strain energies are ∼37 kJ mol^−1^ larger, reflecting that the QM-optimized structure has a lower QM energy than QM/MM-optimized structures. At first, it might be a bit unexpected that there still is some variation in the strain energies depending on the starting structure for the reference-energy calculations. However, this reflects the fixation of the junction H_L_ atoms at different positions. These differences lead to a variation of up to 0.06 Å in the key distances in the optimized structure.

For the general use of the strain energies, we recommend a QM-optimized structure with fixed H_L_ junctions (described in the previous paragraph), starting from the original (deposited) crystal structure, which would ensure that the junction atoms reside at the same position for the various interpretations of the structure.

### 3.4. Application on V-nitro­genase

Next, we try to reproduce the results of a previous application of *ComQum-X* on the active-site FeV cluster of vanadium nitro­genase and in particular the nature of the bidentate ligand (Bergmann *et al.*, 2021*c* ▸). Nitro­genase is the only enzyme that can cleave the triple bond in N_2_ to form two molecules of ammonia (Seefeldt *et al.*, 2020 ▸). There are three variants of nitro­genase, depending on the metal composition of the active site (Jasniewski *et al.*, 2020 ▸). The crystal structures are known for all three variants (Einsle & Rees, 2020 ▸; Trncik *et al.*, 2023 ▸; Sippel & Einsle, 2017 ▸). The most common and most active one is Mo-nitro­genase, which contains an MoFe_7_S_9_C cluster in the active site. In Fe- and V-nitro­genase, Mo is replaced by Fe and V, respectively. However, in the latter case, one of the sulfide ions is also replaced by a bidentate ligand. From the crystallographic raw data, it was not possible to settle the nature of the bidentate ligand; carbonate or nitrate were possible interpretations (Sippel & Einsle, 2017 ▸). In 2021, we published a quantum-refinement study, in which we re-refined the crystal structure of V-nitro­genase with either 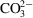, 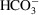 or 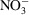, and showed that 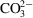 fitted the experimental data best (Bergmann *et al.*, 2021*c* ▸).

Here, we have repeated the calculations with the new *QRef* implementation within *Phenix*. The quantum-refinement calculations were based on the 5n6y crystal structure, obtained at 1.35 Å resolution (Sippel & Einsle, 2017 ▸). As the quantum system, we used the full FeV cluster from the A subunit of the protein (VFe_7_S_8_C), the bidentate ligand, homocitrate, the side chains of Lys83A and Lys362A (modelled as CH_3_NH_3_^+^), the imidazole ring of His423A, the side chain of Cys257A (CH_3_S^−^), as well as the whole Arg339A (except O, but including a –COCH_3_ group from Pro338A), and the side chain of Thr335A (modelled by CH_3_OH). The positively charged Arg and Lys residues were included to compensate the high negative charge of the FeV cluster (Cao *et al.*, 2020 ▸). The backbone NH group of Arg339A also donates a hydrogen bond to the bidentate ligand. This QM system is illustrated in Fig. 2 ▸(*b*). We studied it in the resting *E*_0_ state, in the open-shell singlet state with the oxidation-state assignment V(III)­Fe(II)_3_Fe(III)_4_ (Yang *et al.*, 2021 ▸) and used the broken-symmetry (BS) BS-235 state (*i.e.* all Fe ions were in the high-spin state with a surplus of β spin on Fe2, Fe3 and Fe5) (Bergmann *et al.*, 2021*c* ▸; Benediktsson & Bjornsson, 2020 ▸). We tested three different interpretations of the unknown bidentate ligand: 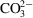, giving a net charge for the whole QM system of −2, 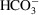 or 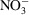, both giving a net charge of −1.

We first performed an investigation to settle the proper value of the *w*_x_ weight factor. The results in Table S3 and Fig. S2 show that *w*_x_ = 3 seems to be a proper compromise between the strain energies and RSZD factors. In Table 3 ▸, we compare the results obtained with the *ComQum-X* and *QRef* implementations. It can be seen that the three crystallographic quality measures are similar or slightly improved (especially RSZD) with *QRef* compared with the *ComQum-X* results. On the other hand, the strain energy (Δ*E*_QM1_ in Table 3 ▸) is higher than in the previous study because it was calculated with another reference in this study.

Most importantly, it is still clear that 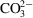 fits the experimental raw data best. In fact, all quality measures for the bidentate ligand are best for 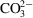. The difference is largest for RSZD, which is 0.6 for 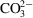 and 1.1 for the two other ligands. However, the strain energy is slightly lower for 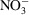 (193 kJ mol^−1^) than for 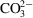 (209 kJ mol^−1^), but this is expected because the net charge of 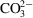 is larger than for the other two ligands (–1), making electrostatic energies larger (Bergmann *et al.*, 2021*a* ▸).

The conclusion that 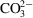 is the correct ligand is also supported by the electron-density difference maps in Fig. S3, which show that there are slightly fewer features in the difference maps for 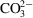 than for the other two ligands, although the differences are quite small. Thus, we can conclude that *QRef* works well and reproduces the results obtained with *ComQum-X*. Moreover, the lower RSZD scores and better difference maps indicate that *QRef* does a slightly better job than *ComQum-X* for the complicated FeV cluster in nitro­genase.

### 3.5. Application on Fe-nitro­genase

We have also run *QRef* calculations on another X-ray crystal structure, *viz.* on the recent structure of Fe-nitro­genase (8boq) at 1.55 Å resolution (Trncik *et al.*, 2023 ▸). The QM model was Fe_8_S_9_C(homocitrate)(SCH_3_)(CH_3_-imidazole) [shown in Fig. 2 ▸(*c*)], where the latter two groups are models of Cys257A and His423A from the A subunit of the enzyme. The QM system was studied in the open-shell singlet state using the BS-2358 state [*i.e.* Fe2, Fe3, Fe5 and Fe8 have a surplus of β spin, whereas the other four Fe ions have a surplus of α spin (Jiang *et al.*, 2023 ▸)]. The original crystal structure involves disorder in the S2B atom of the FeFe cluster (modelled as 50% S2B and 50% as an O atom). However, in our calculations, the occupancy of S2B was set to 1.00 and the oxygen atom was discarded. Gln176A was modelled with dual conformations as in the PDB file.

**Figure 4:**
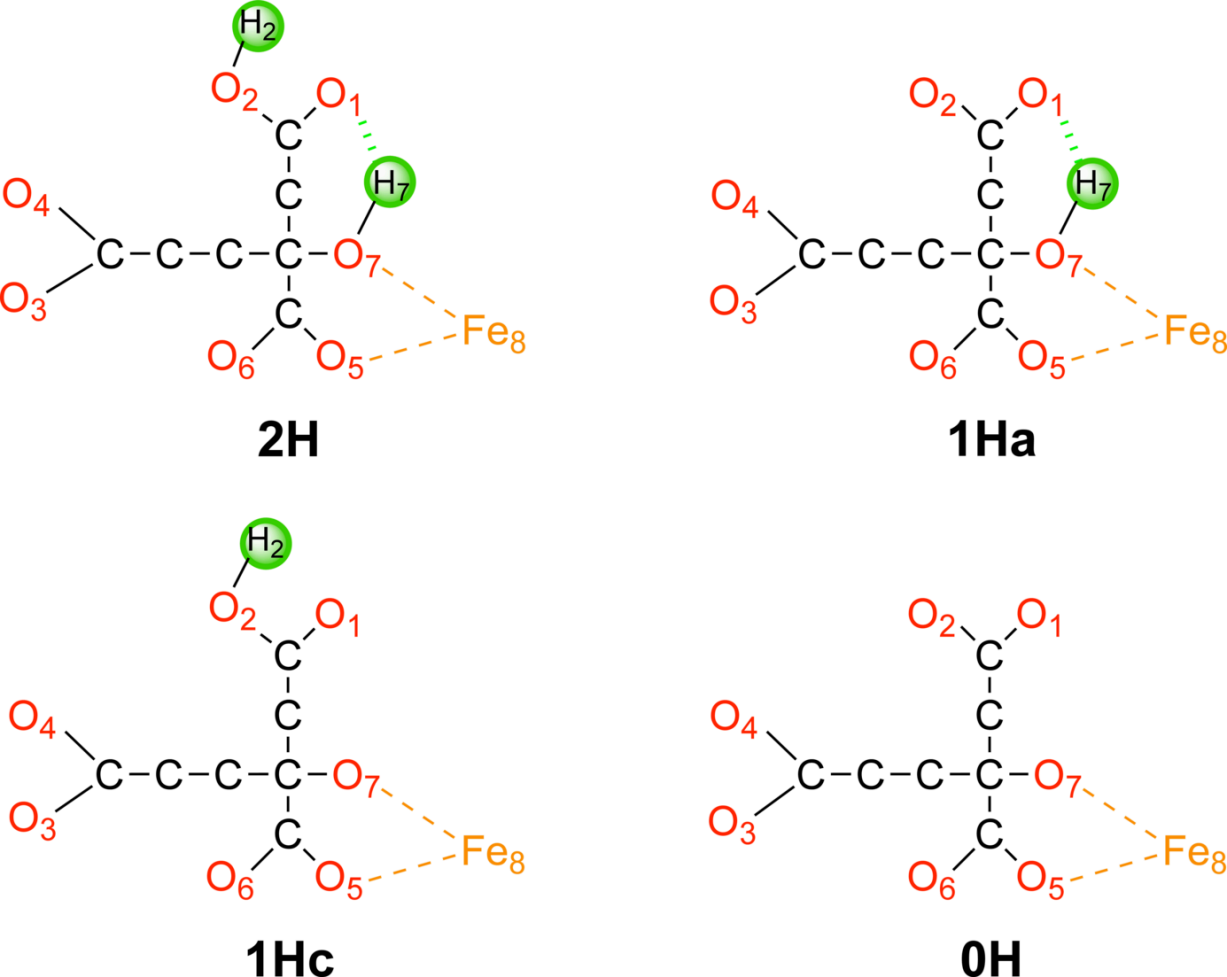
The four protonation states of homocitrate considered: 2H, 1Ha, 1Hc and 0H. Atom numbers are also shown. Non-polar H atoms have been omitted. The charges of homocitrate are −2, −3, −3 and −4, respectively, in these four protonation states.

In fact, these calculations (performed with *QRef*) have already been published (Jiang *et al.*, 2023 ▸) in a different context. The active site of this protein contains an Fe_8_S_9_C cluster, similar to that of V- and Mo-nitro­genase, but with Mo or V replaced by Fe. Similar to the other two nitro­genases, it contains a homocitrate molecule, which forms a bidentate coordination to Fe. Homocitrate contains three carboxyl­ate groups and one alcohol group. The latter and one carboxyl­ate O atom coordinate to the metal. An examination of the hydrogen bonds in the crystal structure indicated that only one of the carboxyl­ate atoms (O2) may be protonated (in addition to the alcohol atom, O7). Therefore, there are four possible protonation states of the homocitrate ligand, with no, one (on either O2 or O7) or two protons, as is shown in Fig. 4 ▸. Previous quantum-refinement calculations have shown that in Mo-nitro­genase, the homocitrate ligand has one proton that is shared between the alcohol O atom and a carboxyl­ate atom (the 1Ha structure). The new quantum-refinement calculations (Jiang *et al.*, 2023 ▸) showed that this is also the case for Fe-nitro­genase.

Here, we will mainly discuss how the results vary with the weight factors. The results are collected in Table 4 ▸. First, we allowed *Phenix* to automatically select the *w*_x_ weight factor (with *w*_c_ = 1). This is done individually for each macrocycle of the refinement and for each protonation state, so the calculations are not fully comparable (the strain energy strongly depends on the weight factors). On average, *w*_x_ was ∼9 for the last two macrocycles. This gave rather large strain energies of 120–168 kJ mol^−1^. It also gave structures that varied little with the protonation state of homocitrate. For example, the Fe—O7 distance to the alcohol atom of homocitrate was 2.17–2.19 Å in the four structures, although O7 is deprotonated in the 0H and 1Hc structures, but protonated in the other two structures; in the corresponding QM/MM structures, this bond length varies from 1.99 Å for 0H to 2.31 Å for 2H. This indicates that *q*_w_ = *w*_x_/*w*_c_ is too high. Similar but more comparable results are obtained if *w*_x_ is explicitly set to 9 in the calculations.

If we instead set *q*_w_ = 0.1, we get very small strain energies (0–7 kJ mol^−1^) and the structures are nearly identical to the corresponding QM/MM structures (within 0.01 Å for the Fe—O distances). This indicates that *q*_w_ is too low and we simply obtain QM/MM structures with no influence from the crystallography.

However, for intermediate values of *q*_w_, we obtain structures that are a compromise between crystallography and QM. We studied two such systems, *viz.* one with *w*_x_ = 1 and *w*_c_ = 1 (*q*_w_ = 1) and one with *w*_x_ = 9 and *w*_c_ = 10 (*q*_w_ = 0.9), to illustrate that what matters is the *w*_x_/*w*_c_ quotient. The two sets of calculations provide similar results with strain energies of 33–54 kJ mol^−1^ and average deviation of the two Fe—O bond lengths from the corresponding QM/MM structures (Δ*d*_av_) of 0.02–0.05 Å. We will use these two sets of calculations for the remaining discussion.

To decide which of the four protonation states fit the crystallographic raw data best, we studied several quality measures. First, we looked at RSZD, RSR and RSCC of the homocitrate ligand to show how well the various models fit the experimental electron density. From Table 4 ▸, it can be seen that all three measures highlight 1Ha as the best protonation state (*i.e.* with the alcohol atom protonated and the carboxyl­ate group deprotonated). In particular, RSZD is 0.5 for this protonation state, whereas it is 1.0–2.8 for the other protonation states. 1Ha is also the protonation state that gives the lowest Δ*d*_av_, 0.01–0.03 Å lower than for the other protonation states. However, the strain energy is lower for the 0H or 2H states by 5–7 kJ mol^−1^. The reason for this is that the strain energy also depends on the net charge of the QM system and therefore the strain energies are fully comparable only for the 1Hc and 1Ha states, for which 1Ha is always lower, but not between the other protonation states.

In conclusion, the quantum-refinement calculations show that 1Ha is the most likely protonation state, which is in agreement with QM/MM calculations (Jiang *et al.*, 2023 ▸) and with the corresponding results obtained for Mo-nitro­genase (Cao *et al.*, 2017 ▸; Benediktsson & Bjornsson, 2017 ▸). Moreover, they point out a practical procedure to determine a proper value of *q*_w_ for the quantum-refinement calculations: *q*_w_ should be selected so that the structures are influenced by both the experimental and the QM data, *i.e.* so that the strain energies are reasonable (10–200 kJ mol^−1^, although the absolute value depends on the size of the QM region, the net charge and the definition of the reference state) and that the geometries depend on the protonation state (or other variations of the composition) and are not identical to the corresponding QM/MM structure. The exact value of *q*_w_ can be adapted to bias the structure slightly towards experiments or QM, depending on the accuracy (resolution) of the experimental data.

### 3.6. Application on a cryo-EM structure of pMMO

Finally, we also tested *QRef* on a cryo-EM structure. Recently, Rosenzweig and coworkers have published eight cryo-EM structures of pMMO from different sources and in different membrane-like surroundings (Koo *et al.*, 2022 ▸). We studied the Cu_D_ site in the pMMO structure 7s4h at 2.14 Å resolution (Koo *et al.*, 2022 ▸). In the deposited PDB file, the Cu ion is three-coordinate, with bonds to the side chains of Asn227C, His231C and His245C. The structure is somewhat pyramidal. The Cu—O distance is 2.21 Å and one of the Cu—N distances is 2.00 Å. However, the other Cu—N distance is quite unrealistic, 1.50 Å. Two water molecules (HOH406C and 415C) are relatively close to the Cu site, but at non-bonding distances of 3.3 and 3.8 Å. However, together with the two coordinating N atoms of the His ligands, they form a reasonable square plane, with Asn227C in an axial position.

In this study, we examined how quantum refinement could improve the structure and how the results vary with the *w*_x_ weight factor (keeping *w*_c_ at 1; in fact, in real-space refinement in *Phenix*, there is only one weight factor that can be varied, *w*_x_) and with the size of the QM region. The Cu ion was always considered in the Cu(I) state [a more thorough investigation of the actual nature of this and the other Cu sites in the recent cryo-EM structures (Chang *et al.*, 2021 ▸; Koo *et al.*, 2022 ▸) will be performed separately]. As the experimental quality measure, we employ the RSCC of the residues in the QM system, calculated using *phenix.map_model_cc*.

The real-space refinement of cryo-EM structures in *Phenix* is approximate in that it does not consider the difference between the experimental and calculated electrostatic potential (ESP) maps over the entire volume of the protein, but only the experimental value of the ESP at the atomic positions (Afonine *et al.*, 2018 ▸). Moreover, the ESP map is typically sharpened so that the ESP becomes almost a square potential (Afonine *et al.*, 2018 ▸). As an effect, the structure depends strongly on the empirical restraints to obtain a chemically reasonable structure. If no restraints are employed, atoms tend to implode into the centre of local density and quality measures such as RSCC will deteriorate, because they are calculated in the proper way, *viz.* comparing the experimental and calculated ESPs in a volume.

This can be seen from the results in Table S4, where *w*_x_ is varied in standard real-space cryo-EM refinement of pMMO with *Phenix* (*i.e.* without QM). It can be seen that RSCC shows a small improvement as *w*_x_ is increased from 0 to 100, from 0.82 to 0.86 on average for the ten considered residues. With the automatic selection of *w*_x_, giving a final value of *w*_x_ = 4.4, an intermediate RSCC score is obtained, 0.84, which is identical to that obtained from the deposited structure (all individual RSCC values are within 0.01, except that for HOH415C, for which RSCC of the re-refined structure is considerably better than in the deposited structure, 0.76, versus 0.67. However, the Cu–ligand distances are very different, *e.g.* 1.95 versus 1.50 Å for Cu—N_His245_ and 3.16 versus 3.81 Å for Cu—O_W406_). Thus, RSCC is quite indifferent to the detailed geometry of the Cu site. For *w*_x_ = 1000, RSCC deteriorates slightly and for *w*_x_ = 10000 it becomes worse than in any other refinement (0.78 on average) and the atoms start to crowd up. This behaviour should be remembered when we run quantum refinement with different values of *w*_x_.

We then performed a set of quantum-refinement calculations with only Cu^+^ and the three strong ligands in the QM system {Asn227C, His231C and His245C, modelled as [Cu(imidazole)_2_(CH_3_CONH_2_)]^+^}. The results are collected in the upper third of Table 5 ▸. It can be seen that for *w*_x_ ≤ 10, the results are very similar. The two Cu—N bond lengths are 1.90–1.92 Å, whereas the weaker Cu—O_Asn227_ bond is 2.08–2.10 Å. Thus, the quantum refinement directly corrects the unrealistically short Cu—N bond length to His245C in the deposited cryo-EM structure (1.50 Å). Likewise, the strain energy (compared with a vacuum-optimized structure with H-link atoms fixed) is small, 19–21 kJ mol^−1^. The average RSCC for the four optimized residues is slightly worse than in the deposited structure, 0.86, compared with 0.89. However, for *w*_x_ = 30, the strain energy starts increase (to 38 kJ mol^−1^) and the Cu–ligand bonds show a larger variation. For *w*_x_ = 100, the strain energy is 100 kJ mol^−1^ and the Cu—O_Asn_ bond has increased to 2.53 Å. At higher *w*_x_ values, the structure breaks down. Interestingly, RSCC decreases significantly for all structures with *w*_x_ ≥ 10, 0.77–0.84. This indicates that the QM potential is less effective than the MM potential in avoiding a structure collapse caused by the approximate treatment of the ESP map in the real-space refinement (the MM bond potential increases monotonically when the bond is stretched, whereas the QM potential allows bonds to break).

Next, we added two water molecules to the QM region and reran the quantum refinement. The results are shown in the middle third of Table 5 ▸. In the quantum-refinement calculations with *w*_x_ ≤ 1, both water molecules come much closer to the Cu ion, with Cu—O distances of 2.52–2.54 Å for one and 2.88–2.92 Å for the other. This is in accordance with EXAFS and ENDOR spectroscopy, identifying a water ligand for the Cu_D_ site (Cutsail *et al.*, 2021 ▸; Jodts *et al.*, 2021 ▸). It is also expected because no electrostatics are included in the cryo-EM refinement, whereas it is explicit in the QM calculations. Therefore, there are no competing interactions in the quantum-refinement calculations from the surroundings and the water molecules always gain some energy by interacting with the Cu ion [even if Cu(I) normally prefers 2–4-coordinate structures]. The strain energies are 37–39 kJ mol^−1^. The Cu—O_Asn227_ distance and the two Cu—N distances are slightly longer than in the structures without water molecules, 2.11–2.13, 1.93–1.94 and 1.97–1.98 Å. The average RSCC of the six residues in the QM system is slightly lower than for the deposited structure, 0.80 compared with 0.83. However, even if the water molecules have moved quite far from their original positions, the RSCC of one of the water molecules decreases only slightly (from 0.71 to 0.68), whereas that of the other water actually increases (from 0.67 to 0.71–0.72). When *w*_x_ is increased, the structure changes slightly and from *w*_x_ = 30, strain energies increase and RSCC decreases. The Cu—O_Asn227_ and Cu—O_W415_ distances increase, whereas the Cu—O_W406_ distance first decreases before it increases.

Finally, we also tested an even larger system, in which we included four neighbouring residues: Asp156C, Arg165C and His173C [modelled as acetate, methyl­guanidinium and methyl­imidazole; shown in Fig. 2 ▸(*d*)] all form hydrogen bonds to the more distant water molecule in the deposited structure (HOH406C), whereas the other water molecule (HOH415C) is too distant from other groups to form any good hydrogen bonds (3.6 Å to Asp156C and 3.9 Å to the first water molecule). We also included Phe177C (modelled as phenol) which restricts the movement of the two water molecules. These calculations were run with a conductor-like polarized continuum model (CPCM) continuum solvent (Cammi *et al.*, 2000 ▸) with a dielectric constant of 4 to improve convergence and to avoid spurious proton transfers within the QM system (Bergmann *et al.*, 2021*a* ▸). The results (shown in the lower third of Table 5 ▸) are similar to those of the other two sets of calculations.

For *w*_x_ ≤ 3, one water molecule (HOH415C without any hydrogen-bonded network) coordinates to Cu at a Cu—O distance of 2.06–2.15 Å. This leads to an elongation of the distance to Asn227 (Cu—O = 2.25–2.26 Å) and the two Cu—N distances (1.93–1.96 Å and 2.02–2.05 Å). The other water molecule (HOH406C) forms strong hydrogen bonds to Asp156C, Arg165C and His173C and resides at a Cu—O distance of 3.28–3.30 Å. The average RSCC over the ten residues in the QM region is 0.81, slightly lower than for the deposited structure (0.84). The strain energy is larger than for the smaller QM systems (reflecting that the QM system is larger, so that more atoms can be strained) and more variable, 93–99 kJ mol^−1^. This reflects that the structure is flexible and contains many strong hydrogen bonds involving several charged groups. In fact, for *w*_x_ = 10, a slightly different structure (local minimum) is obtained, involving an even shorter Cu–O_W415_ bond (2.02 Å) and a longer Cu—O_W406_ distance (2.53 Å). It has a slightly lower strain energy (85 kJ mol^−1^), showing that it is closer to the ideal QM structure. Many more local minima can be obtained by starting from other structures. For *w*_x_ > 10, the structure changes more, RSCC decreases and the strain energy increases.

Based on these results, we recommend that the *w*_x_ weight factor is selected at the point just before RSCC and the strain energy starts to increase, *viz.w*_x_ = 3 in our test case [*cf*. Fig. S4(*c*)]. The quantum refinement corrects the strange Cu—N_His245_ bond length in the original structure and indicates that the Cu_D_ site probably involves one coordinated water molecule.

Note that the real-space cryo-EM refinement is very sensitive to the starting structure. The results in Table 5 ▸ were obtained by starting from one quantum-refined structure for each size of the QM system. If the refinement was instead started from the deposited structure (which is less similar to the quantum-refined structures), much larger variations in the strain energies are obtained, even for structures with *w*_x_ = 0–1, *viz.* 4–5 (two smallest QM regions) and 68 kJ mol^−1^ (largest QM region), reflecting that the systems have converged to different local minima (in particular regarding the hydrogen-bonded pattern of the two water molecules). This reflects that the density map presents a flat potential with little preference for different detailed structures.

**Figure 5:**
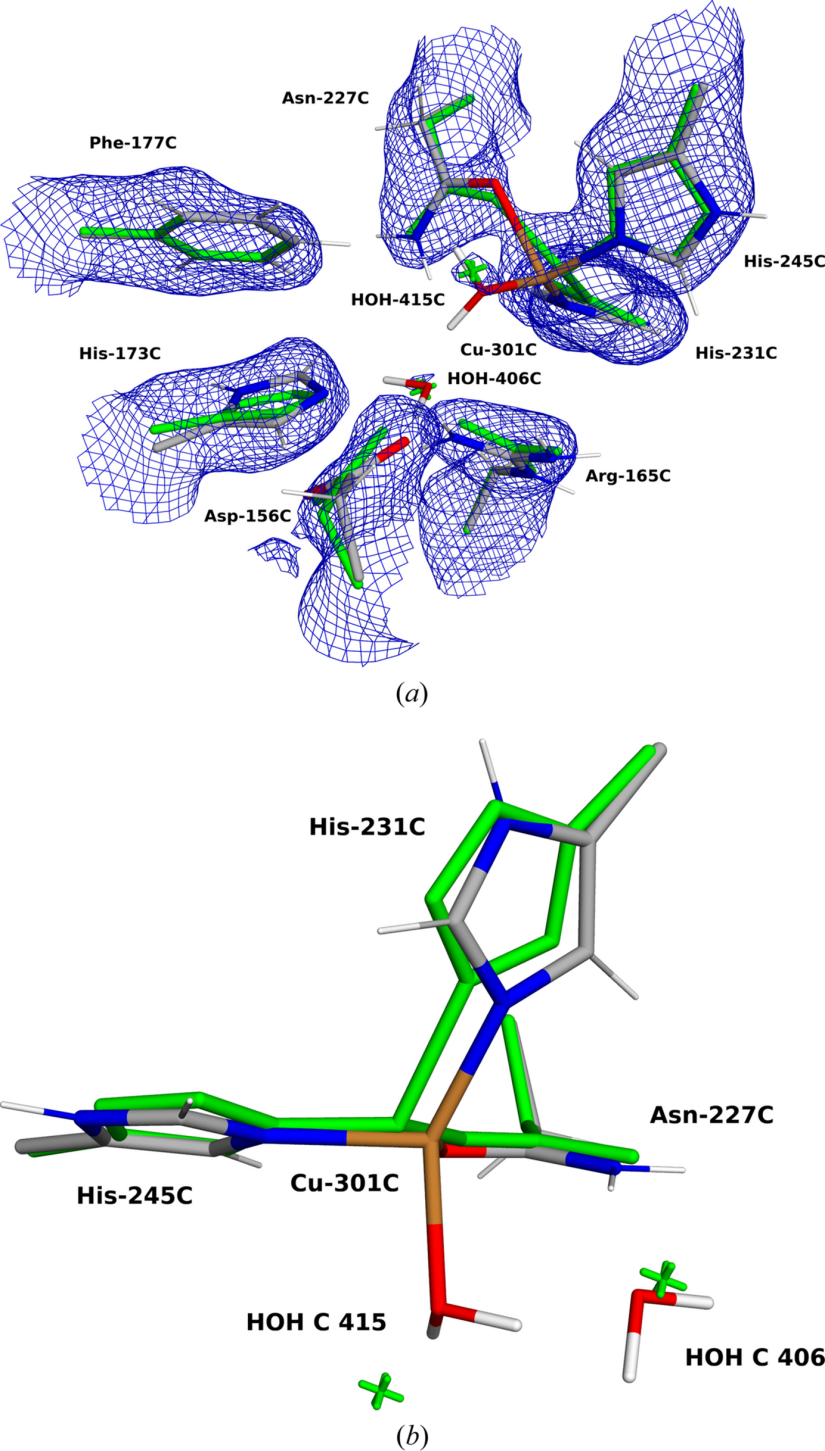
(*a*) Comparison of the deposited (green) and the quantum-refined (atomic colours; largest QM system with *w*_x_ = 3) structures of the Cu_D_ site in pMMO, together with the 2*mF*_o_ − *DF*_c_ map at 0.7σ. (*b*) Another projection emphasizing the change in the Cu—N distance to His245C.

In Fig. 5 ▸, we compare the deposited and quantum-refined structures (the latter obtained with the largest QM system) of the Cu_D_ site, including also the ESP map. It can be seen that the two water molecules give quite weak features in the map (seen only at 0.7σ; only two water molecules in the entire structure have a lower ESP value at the O atom than HOH406C). In the deposited structure, the water molecules and the Cu ion are located in the centre of the ESP, reflecting that the standard empirical restraints do not affect the water molecules or metals (no electrostatics are employed and van der Waals interactions mainly ensure that atoms do not come in too close contact), so that their positions are determined by the cryo-EM data only. However, in the quantum-refined structures, they are moved out of the ESP to also fit the preferences of the QM calculations, *i.e.* the expected bond lengths of the coordinative bonds to the metal and to optimize the hydrogen-bond interactions. Thus, like the remainder of the cryo-EM structure, the final model reflects a compromise between the experimental density and the chemical expectations, described either by empirical restraints or QM calculations. This is expected and is a desired property of quantum refinement, supplementing the experimental data with prior chemical information of the expected structure also for water molecules and metal sites.

To check that the displacement of the HOH406C and 415C water molecules out of the ESP is not too large and unrealistic, we compared the ESP at each atom in the structure with the largest ESP in a sphere with a radius of 1.0 Å around that atom (ΔESP; to measure the displacement out of the maximum ESP; the largest ESP was estimated by a 485 points grid search and ESPs were estimated from the map by trilinear interpolation). In absolute terms, 49% of the protein atoms have a ΔESP larger than that of the water molecule with the largest ΔESP (after quantum refinement; HOH406C). Thus, the movement of the water molecules out of the ESP is not conspicuous compared with the protein atoms (which are affected by the empirical potential). On the other hand, none of the other water molecules had a ΔESP larger than HOH406C (the largest ΔESP was only 41% of that of HOH406C). This shows that the positions of the water molecules are entirely determined by the ESP map and this most likely gives a rather inaccurate estimate of their true positions compared with the protein atoms, owing to the lack of empirical restraints. This could be improved using electrostatic or QM restraints, as in quantum refinement. The same applies to the Cu ions (the highest ΔESP is 36% of that of HOH406C).

## 4. Conclusions

We present a new implementation of quantum refinement, based on the *ORCA* and *Phenix* software, which are both freely available for academic users. The implementation is available on GitHub (https://github.com/krlun/QRef). It requires only the definition of the QM region and specification of the QM method, as well as the charge and multiplicity of the QM region, in addition to the normal refinement settings.

The interface with *Phenix* opens up for the application of quantum refinement for all experimental methods supported by *Phenix*. In this study, we show four typical applications, involving X-ray and neutron crystallography, as well as cryo-EM structures. We show that we can reproduce quantum-refinement results obtained with our previous CNS-based version, *ComQum-X* (Ryde *et al.*, 2002 ▸), regarding the bidentate ligand in V-nitro­genase (Bergmann *et al.*, 2021*c* ▸). For Fe-nitro­genase and MnSOD, we show applications on X-ray and neutron structures. We show that strain energies and the Δ*d* change in key distances can be used to determine how close the refined structure is to an ideal QM structure and discuss how a proper reference structure should be selected. Likewise, we show that standard local crystallographic measures, such as RSZD scores and electron density difference maps, can be used to evaluate how well the refined structure fits the experimental data.

Moreover, we illustrate that the results depend on the *w*_x_ weight factor in a reasonable and understandable way: for low values of *w*_x_, the structure is biased towards QM or the empirical restraints, so that the strain energy and Δ*d* are low and the RSZD scores are high. For high values of *w*_x_, the structure is biased towards the experimental data, so that RSZD scores are low and the strain energy and Δ*d* are high. The ideal compromise is obtained at intermediate values. For simple applications of quantum refinement, the *w*_x_ value suggested by *Phenix* (without any QM) can be used. However, when comparing different structural interpretations of the QM region (which is a typical use of quantum refinement), it is important to use the same value of *w*_x_ for all refinements, otherwise the strain energies, Δ*d* values, RSZD scores and difference maps are not comparable. A proper choice would be the average value suggested by *Phenix* in the various macrocycles with the final target function (they typically vary by a factor of 2–9 in the various macrocycles). If it is found that the refined structures have large strain energies (>200 kJ mol^−1^) and are not affected by the QM calculations, a scan of *w*_x_ values can be performed as in this study. *w*_x_ can then be selected by plotting the strain energy and the averaged relative RSZD scores versus *w*_x_ [*cf*. Figs. 3 ▸(*a*), S2 and S4]. The ideal *w*_x_ is the one in the middle of the range where both the strain energy and the RSZD have started to increase but have not reached unacceptable ranges (>200 kJ mol^−1^ for the strain and >3 for RSZD).

Finally, we also applied *QRef* to a cryo-EM structure. This is a new area of application of our approach [although an application of Q|R on cryo-EM structures has been presented (Wang *et al.*, 2020 ▸)]. Cryo-EM data are typically at quite a low resolution and therefore the final structure strongly relies on empirical restraints. This works fine for normal protein or nucleic-acid structures, but for metal sites it may be a large problem because it is difficult to design accurate MM methods for metals (Hu & Ryde, 2011 ▸). Consequently, we suggest that quantum refinement could become a standard method to treat metal sites in cryo-EM structures. Our calculations indicate that the *w*_x_ weight factor should be selected at the point just before the strain energy starts to increase and the RSCC score starts to decrease. The metal site should be set up with great care because, owing to the flatness of the map, QM will move all putative ligands to the metal unless competitive interactions are also included in the QM model. In future applications, we will test more metal sites in cryo-EM structures and fine-tune the method.

## Supplementary Material

Coordinates of the best quantum-refined structures. DOI: 10.1107/S2052252524008406/jt5077sup1.zip

Supplementary methods, tables and figures. DOI: 10.1107/S2052252524008406/jt5077sup2.pdf

## Figures and Tables

**Table 1:** Results of quantum refinement with different weight factors for the Mn site in the B subunit of MnSOD The table shows seven key distances (N_1_—N_3_ are the coordinating N atoms of His26B, His74B and His163B), as well as Δ*d*_av_ (the average difference in the seven distances between the quantum-refined structure and the structure used to calculate Δ*E*_QM1_), RSZD averaged over the nine residues in the QM region and the strain energy (Δ*E*_QM1_). The individual RSZD values are given in Table S1.

			Distance to Mn (Å)									
*w*_x_	*w*_c_	*w*_QM_	N_1_	N_2_	O_Asp_	N_3_	O_W_	H_W_—N_Gln_ (Å)	O_W_—H_W_ (Å)	Δ*d*_av_ (Å)	RSZD_av_	Δ*E*_QM1_ (kJ mol^−1^)
0	1	7.5	2.27	2.24	2.02	2.28	1.99	1.12	1.49	0.000	1.2	0
0.01	1	7.5	2.27	2.26	2.02	2.27	1.98	1.09	1.53	0.002	1.2	1
0.03	1	7.5	2.27	2.26	2.02	2.27	1.98	1.09	1.53	0.013	1.2	4
0.1	1	7.5	2.27	2.27	2.02	2.27	1.98	1.09	1.53	0.014	1.2	4
0.3	1	7.5	2.27	2.25	2.03	2.28	1.98	1.10	1.50	0.003	1.2	1
1	1	7.5	2.27	2.26	2.02	2.28	1.99	1.11	1.51	0.005	1.1	2
3	1	7.5	2.31	2.29	2.05	2.27	2.01	1.09	1.63	0.025	0.8	22
10	1	7.5	2.33	2.36	2.09	2.25	2.04	1.09	1.77	0.067	0.8	117
30	1	7.5	2.33	2.54	2.08	2.21	2.04	1.10	2.08	0.152	0.8	442
100	1	7.5	2.23	2.99	1.95	2.05	2.85	1.59	1.17	0.406	0.7	1615
1	0	0	4.21	6.04	3.30	2.33	3.53	1.50	2.07	1.341	0.3	1079359
Deposited			2.10	2.25	2.16	2.21	2.25	1.59	0.97	0.233	1.3	362

**Table 2:** Test of different strain energies and reference structures The results are based on the quantum refinement with different *w*_x_ weight factors (*w*_c_ = 1 and *w*_QM_ = 7.5 for all) for the Mn site in the B subunit of MnSOD shown also in Table 1 ▸. *E*_1_, *E*_2_ and *E*_3_ are the QM energies (in kJ mol^−1^) of the QM region in the quantum-refined structure, the QM/MM-optimized structure (*i.e.* with *w*_x_ = 0, but started from the quantum-refined structures with different *w*_x_) and in the isolated QM region optimized by QM, keeping the H_L_ atoms fixed at the starting quantum-refined structure. Δ*E*_1_ = *E*_1_ − *E*_2_ and Δ*E*_3_ = *E*_1_ − *E*_3_, both calculated for each row. Δ*E*_2_ = *E*_1_ − *E*_2_ also, but using only the lowest value for *E*_2_ (*i.e.* that obtained for *w*_x_ = 0). Δ*d*_2_ and Δ*d*_3_ are the average deviations of seven key distances (those included in Table 1 ▸) of the two sets of reference structures from those obtained with *w*_x_ = 0. The actual distances are shown in Table S2 and the corresponding distances for the quantum-refined structures are shown in Table 1 ▸.

*w*_x_	*E*_1_ (kJ mol^−1^)	*E*_2_ (kJ mol^−1^)	*E*_3_ (kJ mol^−1^)	Δ*E*_1_ (kJ mol^−1^)	Δ*E*_2_ (kJ mol^−1^)	Δ*E*_3_ (kJ mol^−1^)	Δ*d*_2_ (Å)	Δ*d*_3_ (Å)
0	7912123	−7912123	−7912160	0	0	37	0.000	0.000
0.01	−7912120	−7912121	−7912160	1	3	40	0.018	0.013
0.03	−7912119	−7912122	−7912160	4	4	41	0.014	0.015
0.1	−7912119	−7912122	−7912160	4	4	41	0.014	0.015
0.3	−7912122	−7912122	−7912160	1	1	38	0.015	0.015
1	−7912120	−7912122	−7912160	2	3	40	0.014	0.014
3	−7912097	−7912120	−7912158	22	25	60	0.012	0.012
10	−7911999	−7912116	−7912156	117	124	157	0.010	0.001
30	−7911678	−7912120	−7912155	442	445	477	0.009	0.004
100	−7910497	−7912111	−7912156	1615	1626	1660	0.011	0.007

**Table 3:** Quality measures (RSZD, RSCC and RSR) and strain energies (Δ*E*_QM1_) for the quantum-refinement calculations of the FeV cofactor in V-nitro­genase with different interpretations of the bidentate ligand (*X*O_3_), studied with both the new (*QRef*) and the old (*ComQum-X*) versions of quantum refinement The latter results are from our previous study (Bergmann *et al.*, 2021*c* ▸). The former results were obtained with *w*_x_ = 3 (results with other values are shown in Table S3). The best results are marked in bold.

	RSZD		RSCC		RSR		
Ligand	FeV	*X*O_3_	FeV	*X*O_3_	FeV	*X*O_3_	Δ*E*_QM1_
*QRef*							
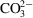	**2.5**	**0.6**	**0.999**	**0.994**	0.012	**0.025**	209
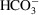	3.0	1.1	**0.999**	0.990	**0.011**	0.031	220
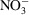	**2.5**	1.1	**0.999**	0.992	0.012	0.028	**193**

*ComQum-X* (Bergmann *et al.*, 2021*c* ▸)							
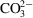	**3.0**	**1.5**	**0.999**	**0.992**	**0.013**	**0.028**	**95.0**
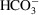	4.0	1.9	**0.999**	0.987	**0.013**	0.034	95.6
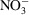	3.9	1.7	**0.999**	0.990	**0.013**	0.029	95.9

**Table 4:** Quality measures and Fe—O distances (Å) for the four quantum-refined structures with varying protonation states of homocitrate (HCA, *cf*. Fig. 4 ▸) and different weight factors The quality measures are RSZD, RSR and RSCC for homocitrate, the strain energy (Δ*E*_QM1_) and the average difference in the Fe—O bond lengths to the homocitrate O5 (carboxyl­ate) and O7 (alcohol) atoms in the quantum-refined structure (Δ*d*_av_), (Fe—O)_QR_, and in a structure optimized by QM/MM, *i.e.* with *w*_x_ = 0, (Fe—O)_QM_. The best results are marked in bold.

									(Fe—O)_QR_		(Fe—O)_QM_	
*w*_x_	*w*_c_	*q*_w_	HCA	RSZD	RSR	RSCC	Δ*E*_QM1_	Δ*d*_av_	O5	O7	O5	O7
Auto	1		0H	**0.6**	**0.042**	0.969	126	0.13	2.25	2.17	2.18	1.99
∼9			1Hc	**0.6**	0.043	0.968	168	0.09	2.26	2.18	2.22	2.05
			1Ha	**0.6**	**0.042**	**0.971**	133	0.10	2.23	2.19	2.13	2.29
			2H	0.7	0.043	0.970	**120**	**0.07**	2.24	2.19	2.22	2.31
9	1	9	0H	0.8	0.043	0.967	172	0.13	2.26	2.16	2.18	1.99
			1Hc	0.8	0.043	0.969	214	**0.07**	2.26	2.18	2.23	2.08
			1Ha	**0.6**	**0.042**	**0.971**	159	0.11	2.23	2.18	2.12	2.29
			2H	**0.6**	0.043	0.970	**156**	0.07	2.25	2.18	2.22	2.30
1	1	1	0H	2.8	0.055	0.944	38	0.05	2.22	2.06	2.19	1.99
			1Hc	1.7	0.050	0.955	54	0.04	2.23	2.13	2.22	2.06
			1Ha	**0.5**	**0.044**	**0.967**	41	**0.03**	2.17	2.20	2.13	2.23
			2H	1.1	0.046	0.962	**35**	0.04	2.22	2.21	2.23	2.28
9	10	0.9	0H	2.6	0.055	0.944	**28**	0.03	2.21	2.04	2.20	1.99
			1Hc	2.3	0.052	0.951	41	0.04	2.23	2.12	2.23	2.04
			1Ha	**0.5**	**0.044**	**0.966**	33	**0.02**	2.16	2.20	2.17	2.24
			2H	1.0	0.046	0.964	35	0.05	2.21	2.21	2.22	2.29
1	10	0.1	0H	5.0	0.054	0.945	**0**	0.00	2.16	1.99	2.16	1.99
			1Hc	4.4	0.063	0.925	7	0.01	2.22	2.07	2.23	2.05
			1Ha	**1.5**	**0.049**	**0.958**	3	**0.00**	2.13	2.25	2.13	2.25
			2H	2.6	0.051	0.950	1	0.01	2.21	2.28	2.21	2.29

**Table 5:** Results of the quantum refinements of the Cu_D_ site of pMMO with three different sizes of the QM system (no water, water or bigger) and different values of the *w*_x_ weight factor (keeping *w*_c_ = 1 and *w*_QM_ = 7.5) The table shows the Cu–ligand distances (in Å) for OD1 of Asn227C (Asn), NE2 of His231C (H1) and His245C (H2), and O of HOH406C (W1) and HOH415C (W2). In addition, the strain energy [Δ*E*_QM1_ in (kJ mol^−1^), relative to the QM system optimized under vacuum with the H_L_ atoms fixed], the average difference in the three or five Cu–ligand distances shown between the quantum-refined structure and the structure used to calculate Δ*E*_QM1_ (Δ*d*_av_), and the average RSCC score for the four, six or ten residues in the QM system are given. Note that the three latter estimates depend on the size of the QM system (therefore different values are given for the deposited structure).

	Distance to Cu (Å)							
*w*_x_	Asn	H1	H2	W1	W2	Δ*E*_QM1_	Δ*d*_av_	RSCC_av_
*No water*								
Deposited	2.21	2.00	1.50			300	0.16	0.89
0	2.09	1.91	1.92			21	0.10	0.86
0.01	2.09	1.91	1.92			21	0.10	0.86
0.03	2.08	1.92	1.92			20	0.11	0.86
0.1	2.09	1.91	1.92			19	0.10	0.86
0.3	2.08	1.91	1.92			20	0.10	0.86
1	2.09	1.90	1.91			21	0.10	0.86
3	2.09	1.91	1.92			21	0.10	0.86
3.6 (auto)	2.10	1.91	1.91			20	0.11	0.86
10	2.10	1.91	1.92			19	0.14	0.85
30	2.02	1.92	1.94			38	0.33	0.84
100	2.53	1.95	1.92			100	0.90	0.83
300	3.33	2.08	1.99			257	1.04	0.80
1000	3.31	2.52	2.07			1812	0.63	0.77

*Water*								
Deposited	2.21	2.00	1.50	3.81	3.30	383	0.39	0.83
0	2.11	1.93	1.97	2.92	2.52	39	0.34	0.80
0.01	2.11	1.93	1.97	2.92	2.52	39	0.34	0.80
0.03	2.11	1.93	1.97	2.92	2.52	39	0.34	0.80
0.1	2.12	1.93	1.97	2.91	2.53	38	0.35	0.80
0.3	2.12	1.93	1.97	2.90	2.53	38	0.35	0.80
1	2.13	1.94	1.97	2.88	2.54	37	0.35	0.80
3	2.13	1.94	1.98	2.87	2.63	35	0.37	0.80
4.9 (auto)	2.12	1.93	1.97	2.86	2.66	30	0.38	0.80
10	2.11	1.94	1.96	2.72	2.81	34	0.44	0.77
30	2.36	2.01	1.95	2.12	3.26	43	0.61	0.69
100	3.45	2.08	1.98	2.04	4.10	148	0.98	0.71
300	3.29	2.15	2.12	2.36	4.49	341	1.00	0.71
1000	3.59	2.49	2.13	3.56	4.79	2046	0.95	0.64

*Bigger*								
Deposited	2.21	2.00	1.50	3.81	3.30	499	0.36	0.84
0	2.25	1.96	2.02	3.29	2.15	99	0.11	0.81
0.01	2.25	1.93	2.04	3.30	2.06	93	0.12	0.81
0.03	2.25	1.95	2.02	3.29	2.14	99	0.11	0.81
0.1	2.25	1.95	2.02	3.29	2.14	98	0.11	0.81
0.3	2.25	1.95	2.04	3.28	2.11	96	0.12	0.81
1	2.25	1.94	2.04	3.28	2.09	95	0.12	0.81
3	2.26	1.93	2.05	3.28	2.06	94	0.13	0.81
5.2 (auto)	2.28	1.92	2.06	3.29	2.03	93	0.14	0.81
10	2.29	1.94	2.06	3.53	2.02	85	0.10	0.81
30	2.44	1.94	2.11	3.72	1.97	105	0.11	0.79
100	2.92	1.0	2.61	3.47	1.88	219	0.38	0.75
300	3.18	1.90	3.21	3.32	1.83	691	0.59	0.70
1000	3.62	2.50	2.11	4.11	2.40	4485	0.54	0.67

## References

[bb1] Adams, P. D., Afonine, P. V., Bunkóczi, G., Chen, V. B., Davis, I. W., Echols, N., Headd, J. J., Hung, L.-W., Kapral, G. J., Grosse-Kunstleve, R. W., McCoy, A. J., Moriarty, N. W., Oeffner, R., Read, R. J., Richardson, D. C., Richardson, J. S., Terwilliger, T. C. & Zwart, P. H. (2010). *Acta Cryst.* D**66**, 213–221. 10.1107/S0907444909052925 PMC2815670 20124702

[bb2] Adams, P. D., Pannu, N. S., Read, R. J. & Brünger, A. T. (1997). *Proc. Natl Acad. Sci. USA*, **94**, 5018–5023. 10.1073/pnas.94.10.5018 PMC24623 9144182

[bb3] Afonine, P. V., Echols, N., Grosse-Kunstleve, R. W., Moriarty, N. W. & Adams, P. D. (2011). *Comput. Crystallogr. Newsl.* **2**, 99–103.

[bb4] Afonine, P. V., Grosse-Kunstleve, R. W., Echols, N., Headd, J. J., Moriarty, N. W., Mustyakimov, M., Terwilliger, T. C., Urzhumtsev, A., Zwart, P. H. & Adams, P. D. (2012). *Acta Cryst.* D**68**, 352–367. 10.1107/S0907444912001308 PMC3322595 22505256

[bb5] Afonine, P. V., Mustyakimov, M., Grosse-Kunstleve, R. W., Moriarty, N. W., Langan, P. & Adams, P. D. (2010). *Acta Cryst.* D**66**, 1153–1163. 10.1107/S0907444910026582 PMC2967420 21041930

[bb6] Afonine, P. V., Poon, B. K., Read, R. J., Sobolev, O. V., Terwilliger, T. C., Urzhumtsev, A. & Adams, P. D. (2018). *Acta Cryst.* D**74**, 531–544. 10.1107/S2059798318006551 PMC6096492 29872004

[bb7] Azadmanesh, J., Lutz, W. E., Coates, L., Weiss, K. L. & Borgstahl, G. E. O. (2021). *Nat. Commun.* **12**, 2079. 10.1038/s41467-021-22290-1 PMC8024262 33824320

[bb8] Benediktsson, B. & Bjornsson, R. (2017). *Inorg. Chem.* **56**, 13417–13429. 10.1021/acs.inorgchem.7b02158 29053260

[bb9] Benediktsson, B. & Bjornsson, R. (2020). *Inorg. Chem.* **59**, 11514–11527. 10.1021/acs.inorgchem.0c01320 PMC7458435 32799489

[bb10] Benediktsson, B. & Bjornsson, R. (2022). *J. Chem. Theory Comput.* **18**, 1437–1457. 10.1021/acs.jctc.1c00753 PMC8908755 35167749

[bb11] Bergmann, J., Oksanen, E. & Ryde, U. (2021*a*). *Acta Cryst.* B**77**, 906–918.

[bb12] Bergmann, J., Oksanen, E. & Ryde, U. (2021*b*). *J. Biol. Inorg. Chem.* **26**, 341–353. 10.1007/s00775-021-01858-8 PMC8068654 33713183

[bb13] Bergmann, J., Oksanen, E. & Ryde, U. (2021*c*). *J. Inorg. Biochem.* **219**, 111426. 10.1016/j.jinorgbio.2021.111426 33756394

[bb14] Bergmann, J., Oksanen, E. & Ryde, U. (2022). *Curr. Opin. Struct. Biol.* **72**, 18–26. 10.1016/j.sbi.2021.07.002 34392061

[bb15] Berman, H. M., Westbrook, J., Feng, Z., Gilliland, G., Bhat, T. N., Weissig, H., Shindyalov, I. N. & Bourne, P. E. (2000). *Nucleic Acids Res.* **28**, 235–242. 10.1093/nar/28.1.235 PMC102472 10592235

[bb16] Blakeley, M. P. (2009). *Crystallogr. Rev.* **15**, 157–218.

[bb17] Borbulevych, O., Martin, R. I., Tickle, I. J. & Westerhoff, L. M. (2016). *Acta Cryst.* D**72**, 586–598. 10.1107/S2059798316002837 PMC4822566 27050137

[bb18] Borbulevych, O. Y., Plumley, J. A., Martin, R. I., Merz, K. M. & Westerhoff, L. M. (2014). *Acta Cryst.* D**70**, 1233–1247. 10.1107/S1399004714002260 PMC4014119 24816093

[bb19] Brunger, A. T. (2007). *Nat. Protoc.* **2**, 2728–2733. 10.1038/nprot.2007.406 18007608

[bb20] Brünger, A. T., Adams, P. D., Clore, G. M., DeLano, W. L., Gros, P., Grosse-Kunstleve, R. W., Jiang, J.-S., Kuszewski, J., Nilges, M., Pannu, N. S., Read, R. J., Rice, L. M., Simonson, T. & Warren, G. L. (1998). *Acta Cryst.* D**54**, 905–921. 10.1107/s0907444998003254 9757107

[bb21] Brünger, A. T., Karplus, M. & Petsko, G. A. (1989). *Acta Cryst.* A**45**, 50–61.

[bb22] Brünger, A. T. & Rice, L. M. (1997). *Methods Enzymol.* **277**, 243–269. 10.1016/s0076-6879(97)77015-0 18488313

[bb23] Burgess, J. (1978). *Metal Ions in Solution.* New York: John Wiley.

[bb24] Caldararu, O., Manzoni, F., Oksanen, E., Logan, D. T. & Ryde, U. (2019). *Acta Cryst.* D**75**, 368–380. 10.1107/S205979831900175X PMC6465982 30988254

[bb25] Caldeweyher, E., Ehlert, S., Hansen, A., Neugebauer, H., Spicher, S., Bannwarth, C. & Grimme, S. (2019). *J. Chem. Phys.* **150**, 154122. 10.1063/1.5090222 31005066

[bb26] Cammi, R., Mennucci, B. & Tomasi, J. (2000). *J. Phys. Chem. A*, **104**, 5631–5637.

[bb27] Cao, L., Caldararu, O. & Ryde, U. (2017). *J. Phys. Chem. B*, **121**, 8242–8262. 10.1021/acs.jpcb.7b02714 28783353

[bb28] Cao, L., Caldararu, O. & Ryde, U. (2018). *J. Chem. Theory Comput.* **14**, 6653–6678. 10.1021/acs.jctc.8b00778 30354152

[bb29] Cao, L., Caldararu, O. & Ryde, U. (2020). *J. Biol. Inorg. Chem.* **25**, 847–861. 10.1007/s00775-020-01813-z PMC7511287 32856107

[bb30] Cao, L. & Ryde, U. (2018). *Front. Chem.* **6**, 89. 10.3389/fchem.2018.00089 PMC5891596 29666794

[bb31] Cao, L. & Ryde, U. (2020). *Acta Cryst.* D**76**, 1145–1156. 10.1107/S2059798320012917 PMC7604908 33135685

[bb32] Chang, W.-H., Lin, H.-H., Tsai, I.-K., Huang, S.-H., Chung, S.-C., Tu, I.-P., Yu, S. S.-F. & Chan, S.-I. (2021). *J. Am. Chem. Soc.* **143**, 9922–9932. 10.1021/jacs.1c04082 34170126

[bb33] Chung, L. W., Sameera, W. M. C., Ramozzi, R., Page, A. J., Hatanaka, M., Petrova, G. P., Harris, T. V., Li, X., Ke, Z., Liu, F., Li, H. B., Ding, L. & Morokuma, K. (2015). *Chem. Rev.* **115**, 5678–5796. 10.1021/cr5004419 25853797

[bb34] Cutsail, G. E., Ross, M. O., Rosenzweig, A. C. & DeBeer, S. (2021). *Chem. Sci.* **12**, 6194–6209. 10.1039/d1sc00676b PMC8098663 33996018

[bb35] Einsle, O. & Rees, D. C. (2020). *Chem. Rev.* **120**, 4969–5004. 10.1021/acs.chemrev.0c00067 PMC8606229 32538623

[bb36] Engh, R. A. & Huber, R. (1991). *Acta Cryst.* A**47**, 392–400.

[bb37] Engh, R. A. & Huber, R. (2012). *International Tables for Crystallography*, Vol. F, pp. 474–484. Chichester: John Wiley & Sons.

[bb38] Fadel, F., Zhao, Y., Cachau, R., Cousido-Siah, A., Ruiz, F. X., Harlos, K., Howard, E., Mitschler, A. & Podjarny, A. (2015). *Acta Cryst.* D**71**, 1455–1470. 10.1107/S139900471500783X 26143917

[bb39] Fields, B. A., Bartsch, H. H., Bartunik, H. D., Cordes, F., Guss, J. M. & Freeman, H. C. (1994). *Acta Cryst.* D**50**, 709–730. 10.1107/S0907444994003021 15299368

[bb40] Furche, F., Ahlrichs, R., Hättig, C., Klopper, W., Sierka, M. & Weigend, F. (2014). *WIREs Comput. Mol. Sci.* **4**, 91–100.

[bb41] Grosse-Kunstleve, R. W., Sauter, N. K., Moriarty, N. W. & Adams, P. D. (2002). *J. Appl. Cryst.* **35**, 126–136.

[bb42] Han, W. G., Lovell, T. & Noodleman, L. (2002). *Inorg. Chem.* **41**, 205–218. 10.1021/ic010355z 11800609

[bb43] Haynes, W. M. (2016). *CRC Handbook of Chemistry and Physics*, 97th ed. Boca Raton: CRC Press.

[bb44] Headd, J. J., Echols, N., Afonine, P. V., Grosse-Kunstleve, R. W., Chen, V. B., Moriarty, N. W., Richardson, D. C., Richardson, J. S. & Adams, P. D. (2012). *Acta Cryst.* D**68**, 381–390. 10.1107/S0907444911047834 PMC3322597 22505258

[bb45] Henderson, R. (2015). *Arch. Biochem. Biophys.* **581**, 19–24. 10.1016/j.abb.2015.02.036 25796174

[bb46] Hsiao, Y.-W., Drakenberg, T. & Ryde, U. (2005). *J. Biomol. NMR*, **31**, 97–114. 10.1007/s10858-004-6729-7 15772750

[bb47] Hsiao, Y.-W., Sanchez-Garcia, E., Doerr, M. & Thiel, W. (2010). *J. Phys. Chem. B*, **114**, 15413–15423. 10.1021/jp108095n 20977248

[bb48] Hsiao, Y.-W., Tao, Y., Shokes, J. E., Scott, R. A. & Ryde, U. (2006). *Phys. Rev. B*, **74**, 214101.

[bb49] Hu, L. & Ryde, U. (2011). *J. Chem. Theory Comput.* **7**, 2452–2463. 10.1021/ct100725a 26606619

[bb50] Jack, A. & Levitt, M. (1978). *Acta Cryst.* A**34**, 931–935.

[bb51] Jasniewski, A. J., Lee, C. C., Ribbe, M. W. & Hu, Y. (2020). *Chem. Rev.* **120**, 5107–5157. 10.1021/acs.chemrev.9b00704 PMC7491575 32129988

[bb52] Jiang, H., Lundgren, K. J. M. & Ryde, U. (2023). *Inorg. Chem.* **62**, 19433–19445. 10.1021/acs.inorgchem.3c02329 PMC10698722 37987624

[bb53] Jodts, R. J., Ross, M. O., Koo, C. W., Doan, P. E., Rosenzweig, A. C. & Hoffman, B. M. (2021). *J. Am. Chem. Soc.* **143**, 15358–15368. 10.1021/jacs.1c07018 PMC8811777 34498465

[bb54] Kleywegt, G. J. & Jones, T. A. (1997). *Methods Enzymol.* **227**, 208–230. 10.1016/s0076-6879(97)77013-7 18488311

[bb55] Koo, C. W., Tucci, F. J., He, Y. & Rosenzweig, A. C. (2022). *Science*, **375**, 1287–1291. 10.1126/science.abm3282 PMC9357287 35298269

[bb56] Liebschner, D., Afonine, P. V., Baker, M. L., Bunkóczi, G., Chen, V. B., Croll, T. I., Hintze, B., Hung, L.-W., Jain, S., McCoy, A. J., Moriarty, N. W., Oeffner, R. D., Poon, B. K., Prisant, M. G., Read, R. J., Richardson, J. S., Richardson, D. C., Sammito, M. D., Sobolev, O. V., Stockwell, D. H., Terwilliger, T. C., Urzhumtsev, A. G., Videau, L. L., Williams, C. J. & Adams, P. D. (2019). *Acta Cryst.* D**75**, 861–877. 10.1107/S2059798319011471 PMC6778852 31588918

[bb57] Liebschner, D., Afonine, P. V., Poon, B. K., Moriarty, N. W. & Adams, P. D. (2023). *Acta Cryst.* D**79**, 1079–1093. 10.1107/S2059798323008914 PMC10833352 37942718

[bb58] Liebschner, D., Moriarty, N. W., Poon, B. K. & Adams, P. D. (2023). *Acta Cryst.* D**79**, 100–110. 10.1107/S2059798323000025 PMC9912925 36762856

[bb59] Maier, J. A., Martinez, C., Kasavajhala, K., Wickstrom, L., Hauser, K. E. & Simmerling, C. (2015). *J. Chem. Theory Comput.* **11**, 3696–3713. 10.1021/acs.jctc.5b00255 PMC4821407 26574453

[bb60] Maliekal, J., Karapetian, A., Vance, C., Yikilmaz, E., Wu, Q., Jackson, T., Brunold, T. C., Spiro, T. G. & Miller, A. F. (2002). *J. Am. Chem. Soc.* **124**, 15064–15075. 10.1021/ja027319z 12475351

[bb61] Maseras, F. & Morokuma, K. (1995). *J. Comput. Chem.* **16**, 1170–1179.

[bb62] Miller, A. F., Padmakumar, K., Sorkin, D. L., Karapetian, A. & Vance, C. K. (2003). *J. Inorg. Biochem.* **93**, 71–83. 10.1016/s0162-0134(02)00621-9 12538055

[bb63] Moriarty, N. W., Grosse-Kunstleve, R. W. & Adams, P. D. (2009). *Acta Cryst.* D**65**, 1074–1080. 10.1107/S0907444909029436 PMC2748967 19770504

[bb64] Moriarty, N. W., Tronrud, D. E., Adams, P. D. & Karplus, P. A. (2016). *Acta Cryst.* D**72**, 176–179. 10.1107/S2059798315022408 PMC4756615 26894545

[bb65] Neese, F. (2006). *J. Biol. Inorg. Chem.* **11**, 702–711. 10.1007/s00775-006-0138-1 16821037

[bb66] Neese, F., Wennmohs, F., Becker, U. & Riplinger, C. (2020). *J. Chem. Phys.* **152**, 224108. 10.1063/5.0004608 32534543

[bb67] Nilsson, K., Lecerof, D., Sigfridsson, E. & Ryde, U. (2003). *Acta Cryst.* D**59**, 274–289. 10.1107/s0907444902021431 12554938

[bb68] Nilsson, K. & Ryde, U. (2004). *J. Inorg. Biochem.* **98**, 1539–1546. 10.1016/j.jinorgbio.2004.06.006 15337606

[bb69] Nogales, E. (2016). *Nat. Methods*, **13**, 24–27. 10.1038/nmeth.3694 PMC4913480 27110629

[bb70] Orlov, I., Myasnikov, A. G., Andronov, L., Natchiar, S. K., Khatter, H., Beinsteiner, B., Ménétret, J. F., Hazemann, I., Mohideen, K., Tazibt, K., Tabaroni, R., Kratzat, H., Djabeur, N., Bruxelles, T., Raivoniaina, F., Pompeo, L. D., Torchy, M., Billas, I., Urzhumtsev, A. & Klaholz, B. P. (2017). *Biol. Cell*, **109**, 81–93. 10.1111/boc.201600042 27730650

[bb71] Reuter, N., Dejaegere, A., Maigret, B. & Karplus, M. (2000). *J. Phys. Chem. A*, **104**, 1720–1735.

[bb72] Rhodes, G. (2006). *Crystallography Made Crystal Clear.* New York: Academic Press.

[bb73] Rulíšek, L., Jensen, K. P., Lundgren, K. & Ryde, U. (2006). *J. Comput. Chem.* **27**, 1398–1414. 10.1002/jcc.20450 16802319

[bb74] Rulíšek, L. & Ryde, U. (2006). *J. Phys. Chem. B*, **110**, 11511–11518. 10.1021/jp057295t 16771427

[bb75] Ryde, U. (1996). *J. Comput. Aided Mol. Des.* **10**, 153–164. 10.1007/BF00402823 8741019

[bb76] Ryde, U. (2016). *Methods Enzymol.* **577**, 119–158. 10.1016/bs.mie.2016.05.014 27498637

[bb77] Ryde, U. & Nilsson, K. (2003). *J. Am. Chem. Soc.* **125**, 14232–14233. 10.1021/ja0365328 14624544

[bb78] Ryde, U., Olsen, L. & Nilsson, K. (2002). *J. Comput. Chem.* **23**, 1058–1070. 10.1002/jcc.10093 12116392

[bb79] Schäfer, A., Horn, H. & Ahlrichs, R. (1992). *J. Chem. Phys.* **97**, 2571–2577.

[bb80] Schröder, G. C. & Meilleur, F. (2021). *Acta Cryst.* D**77**, 1251–1269. 10.1107/S2059798321009025 PMC8489226 34605429

[bb81] Seefeldt, L. C., Yang, Z.-Y., Lukoyanov, D. A., Harris, D. F., Dean, D. R., Raugei, S. & Hoffman, B. M. (2020). *Chem. Rev.* **120**, 5082–5106. 10.1021/acs.chemrev.9b00556 PMC7703680 32176472

[bb82] Senn, H. M. & Thiel, W. (2009). *Angew. Chem. Int. Ed.* **48**, 1198–1229. 10.1002/anie.200802019 19173328

[bb83] Sippel, D. & Einsle, O. (2017). *Nat. Chem. Biol.* **13**, 956–960. 10.1038/nchembio.2428 PMC5563456 28692069

[bb84] Söderhjelm, P. & Ryde, U. (2006). *J. Mol. Struct. Theochem*, **770**, 199–219.

[bb85] Tao, J., Perdew, J. P., Staroverov, V. N. & Scuseria, G. E. (2003). *Phys. Rev. Lett.* **91**, 146401. 10.1103/PhysRevLett.91.146401 14611541

[bb86] Tickle, I. J. (2012). *Acta Cryst.* D**68**, 454–467. 10.1107/S0907444911035918 PMC3322605 22505266

[bb87] Trncik, C., Detemple, F. & Einsle, O. (2023). *Nat. Catal.* **6**, 415–424.

[bb88] Urzhumtsev, A. G. & Lunin, V. Y. (2019). *Crystallogr. Rev.* **25**, 164–262.

[bb89] Wang, L., Kruse, H., Sobolev, O. V., Moriarty, N. W., Waller, M. P., Afonine, P. V. & Biczysko, M. (2020). *Acta Cryst.* D**76**, 1184–1191. 10.1107/S2059798320013194 33263324

[bb90] Wang, Y., Kruse, H., Moriarty, N. W., Waller, M. P., Afonine, P. V. & Biczysko, M. (2023). *Theor. Chem. Acc.* **142**, 100.

[bb91] Yan, Z., Li, X. & Chung, L. W. (2021). *J. Chem. Theory Comput.* **17**, 3783–3796. 10.1021/acs.jctc.1c00148 34032440

[bb92] Yang, Z. Y., Jimenez-Vicente, E., Kallas, H., Lukoyanov, D. A., Yang, H., Martin Del Campo, J. S., Dean, D. R., Hoffman, B. M. & Seefeldt, L. C. (2021). *Chem. Sci.* **12**, 6913–6922. 10.1039/d0sc06561g PMC8153082 34123320

[bb93] Yatsimirksii, K. B. & Vasilev, V. P. (1960). *Instability Constants of Complex Compounds.* Elmsford: Pergamon.

[bb94] Yu, N., Yennawar, H. P. & Merz, K. M. (2005). *Acta Cryst.* D**61**, 322–332. 10.1107/S0907444904033669 15735343

[bb95] Zheng, M., Biczysko, M., Xu, Y., Moriarty, N. W., Kruse, H., Urzhumtsev, A., Waller, M. P. & Afonine, P. V. (2020). *Acta Cryst.* D**76**, 41–50. 10.1107/S2059798319015122 31909742

[bb96] Zheng, M., Moriarty, N. W., Xu, Y., Reimers, J. R., Afonine, P. V. & Waller, M. P. (2017). *Acta Cryst.* D**73**, 1020–1028. 10.1107/S2059798317016746 PMC5713877 29199981

[bb97] Zheng, M., Reimers, J. R., Waller, M. P. & Afonine, P. V. (2017). *Acta Cryst.* D**73**, 45–52. 10.1107/S2059798316019847 PMC5331472 28045384

[bb98] Zundert, G. C. P. van, Moriarty, N. W., Sobolev, O. V., Adams, P. D. & Borrelli, K. W. (2021). *Structure*, **29**, 913–921. 10.1016/j.str.2021.03.011 PMC8349848 33823127

