# Supplementary material for: Quantum refinement in real and reciprocal space using the Phenix and ORCA software
Source: IUCrJ. 2024 Sep 30;11(Pt 6):921–37. doi: 10.1107/S2052252524008406 (PMC11533993; doi:10.1107/S2052252524008406)
Supplement: Supplementary file 2 [file m-11-00921-sup2.pdf]

IUCrJ

Volume 11 (2024)

Supporting information for article:

Quantum refinement in real and reciprocal space using the *Phenix* and *ORCA* software

Kristoffer J. M. Lundgren, Octav Caldararu, Esko Oksanen and Ulf Ryde

Treatment of junctions

As described in the main text, the QRef code interfaces between Phenix and ORCA utilizing a subtractive QM/MM scheme described by

$$E_{\text{restraints}}(\boldsymbol{R}) = w_{\text{QM}} E_{\text{QM1}}(\boldsymbol{R}_{\text{QM1}}) - E_{\text{MM1}}(\boldsymbol{R}) + E_{\text{MM}}(\boldsymbol{R}) \tag{S1}$$

where $\boldsymbol{R}$ is the coordinates for the entire model and $\boldsymbol{R}_{\text{QM1}}$ is the coordinates for the QM subsystem.

Since QM calculations require filled valences, the QM region needs to be truncated (capped) in a proper way. We have used the hydrogen link-atom approach (Ryde, 1996, Reuter *et al.*, 2000), in which the QM region is truncated by adding a hydrogen atom for each QM–MM bond, called the hydrogen link atom (H_L), which typically replaces a carbon atom in the MM region, called the carbon link atom (C_L). The positions of the H_L atoms are unambiguously determined by the positions of the corresponding C_L atoms according to

$$\bar{r}_{\text{H}_\text{L}} = \bar{r}_{\text{X}} + g_{\text{bond}}(\bar{r}_{\text{C}_\text{L}} - \bar{r}_{\text{X}}) \tag{S2}$$

where g_{bond} is a precalculated parameter for each kind of bond, obtained as the quotient between the ideal X–H_L bond distance calculated with the QM method employed for a model system of the truncated residue and the ideal X–C_L bond distance according to the MM force field (Ryde, 1996). In the E_{MM} and E_{MM1} energy calculations, the C_L atoms and positions are used. Eq. S2 is used before each QM calculation on the QM region is performed (i.e. to obtain the E_{QM1} energy).

From Eq. (S1) we can then obtain the gradient relating to the restraints as

$$\nabla E_{\text{restraints}}(\boldsymbol{R}) = w_{\text{QM}} \nabla E_{\text{QM1}}(\boldsymbol{R}_{\text{QM1}}) \cdot J(\boldsymbol{R}_{\text{QM1}}; \boldsymbol{R}) - \nabla E_{\text{MM1}}(\boldsymbol{R}) + \nabla E_{\text{MM}}(\boldsymbol{R}) \tag{S3}$$

where $J(\boldsymbol{R}_{\text{QM1}}; \boldsymbol{R})$ is the Jacobian between the coordinates in the QM subsystem and the coordinates for the entire model (Maseras & Morokuma, 1995, Chung *et al.*, 2015). From equation (S2), we see that a block in $J(\boldsymbol{R}_{\text{QM1}}; \boldsymbol{R})$ relating to a single junction in Cartesian space becomes a 6×6 matrix with the general shape

$$\begin{pmatrix} 1 & 0 & 0 & 0 & 0 & 0 \\ 0 & 1 & 0 & 0 & 0 & 0 \\ 0 & 0 & 1 & 0 & 0 & 0 \\ (1 - g_{\text{bond}}) & 0 & 0 & g_{\text{bond}} & 0 & 0 \\ 0 & (1 - g_{\text{bond}}) & 0 & 0 & g_{\text{bond}} & 0 \\ 0 & 0 & (1 - g_{\text{bond}}) & 0 & 0 & g_{\text{bond}} \end{pmatrix}. \tag{S4}$$

Eq. S3 is used every time the total gradients are calculated.

In QM/MM implementations, QM calculations are performed with H_L atoms, but the E_{MM1} calculations can be performed with either H_L or C_L atoms (Cao & Ryde, 2018). If H_L atoms are used, it opens for the possibility to correct for errors introduced by the use of H_L atoms in the QM calculations. However, this has turned out to be hard in practice and it is error-prone (Cao & Ryde, 2018). In our previous implementation of quantum refinement with CNS (Ryde *et al.*, 2002), we used H_L atoms but aimed only for van der Waals link-atom corrections (Cao & Ryde, 2018), by an automatic procedure that read in the E_{MM} force field and ensured that the bonded terms cancelled. However, this is not possible for improper

dihedrals, the energy of which change in a complicated way if one of the atoms changes from C to H and has a different position. The effect is quite small, but for accurate calculations we simply deleted improper torsions involving H_L also for the E_{MM} term. For this reason and also because the Phenix empirical restraints are more complicated and less accessible, we decided to instead use C_L atoms in the implementation of QRef. In practice, we have shown that the effect of van der Waals link-atom corrections is minor (Cao & Ryde, 2018).

References

Cao, L. & Ryde, U. (2018). *Frontiers in Chemistry* **6**, 89-89.

Chung, L. W., Sameera, W. M. C., Ramozzi, R., Page, A. J., Hatanaka, M., Petrova, G. P., Harris, T. V., Li, X., Ke, Z., Liu, F., Li, H. B., Ding, L. & Morokuma, K. (2015). *Chemical Reviews* **115**, 5678-5796.

Maseras, F. & Morokuma, K. (1995). *Journal of Computational Chemistry* **16**, 1170-1170.

Reuter, N., Dejaegere, A., Maigret, B. & Karplus, M. (2000). *The Journal of Physical Chemistry A* **104**, 1720-1735.

Ryde, U. (1996). *Journal of computer-aided molecular design* **10**, 153-164.

Ryde, U., Olsen, L. & Nilsson, K. (2002). *Journal of Computational Chemistry* **23**, 1058-1070.

Table S1. Residue-wise RSZD values and *R* values for the various quantum refinements with different weight factors for Mn site in the B subunit of MnSOD.

w_x	w_c	w_{QM}	His-26B	Tyr-34B	His-74B	Trp-123B	Gln-143B	Asp-159B	His-163B	Mn	Wat-319	R_{work}	R_{free}
0	1	7.5	0.8	1.4	0.6	1.9	2.0	0.6	0.8	1.2	1.1	0.227	0.304
0.01	1	7.5	1.0	1.4	0.6	2.0	2.0	0.7	0.8	1.2	1.3	0.227	0.304
0.03	1	7.5	0.9	1.5	0.5	2.0	2.0	0.6	0.8	1.2	1.2	0.227	0.305
0.1	1	7.5	1.0	1.4	0.5	2.0	2.0	0.7	0.8	1.2	1.2	0.227	0.304
0.3	1	7.5	0.9	1.4	0.6	2.0	2.0	0.6	0.7	1.2	1.2	0.227	0.304
1	1	7.5	0.7	1.4	0.5	1.9	1.8	0.7	0.8	1.2	1.2	0.227	0.304
3	1	7.5	0.4	1.0	0.2	1.5	0.6	0.7	0.7	1.3	1.2	0.226	0.305
10	1	7.5	0.3	1.1	0.2	1.6	0.2	0.8	0.6	1.5	1.1	0.226	0.305
30	1	7.5	0.2	1.1	0.1	1.6	0.2	0.8	0.7	1.7	1.0	0.226	0.305
100	1	7.5	0.0	1.2	0.3	1.6	0.1	0.7	0.7	0.9	0.5	0.225	0.305
1	0	0	0.1	0.0	0.1	0.9	0.0	0.2	0.1	1.4	0.3	0.224	0.306
deposited			0.7	0.5	1.0	1.7	1.7	0.7	2.5	0.9	1.7	0.250	0.301

Table S2. Key distances (Å) for Mn site in the B subunit of MnSOD (HN = H_w–N_{Gln} and OH = O_W–H_W) obtained in two different reference structures, viz. the QM/MM optimised structure (i.e. with $w_x = 0$) and the isolated QM region optimised by QM, keeping the H_L atoms fixed at the starting quantum-refined structure (giving E_2 and E_3 in Table 2), starting from quantum-refined structures obtained with different values of w_x.

w_x	QM/MM							QM_{opt}						
	N_1	N_2	O_{Asp}	N_3	O_W	HN	OH	N_1	N_2	O_{Asp}	N_3	O_W	HN	OH
0	2.27	2.24	2.02	2.28	1.99	1.12	1.49	2.31	2.23	2.05	2.29	2.00	1.09	1.57
0.01	2.27	2.26	2.02	2.27	1.98	1.09	1.52	2.30	2.23	2.05	2.29	2.00	1.09	1.57
0.03	2.27	2.25	2.03	2.27	1.99	1.11	1.49	2.30	2.24	2.05	2.29	2.00	1.09	1.57
0.1	2.27	2.25	2.03	2.27	1.99	1.11	1.49	2.30	2.24	2.05	2.29	2.00	1.09	1.57
0.3	2.27	2.25	2.02	2.27	1.99	1.11	1.50	2.31	2.23	2.05	2.29	2.00	1.09	1.57
1	2.27	2.25	2.02	2.27	1.99	1.10	1.52	2.31	2.23	2.05	2.29	2.00	1.09	1.57
3	2.30	2.25	2.03	2.27	2.00	1.10	1.56	2.34	2.23	2.05	2.28	2.00	1.09	1.60
10	2.29	2.24	2.03	2.26	2.00	1.09	1.57	2.35	2.23	2.05	2.28	2.01	1.09	1.61
30	2.26	2.23	2.02	2.26	2.00	1.09	1.55	2.36	2.23	2.05	2.28	2.01	1.09	1.63
100	2.27	2.25	2.03	2.26	2.00	1.08	1.58	2.34	2.24	2.06	2.30	2.00	1.09	1.62

Table S3. Quality measures (RSZD, RSCC and RSR) and strain energies (ΔE_{QM1}) for the quantum-refinement calculations of the FeV cofactor in V-nitrogenase with different interpretations of the bidentate ligand and different weight factors.

	w_x	w_c	w_{QM}	RSZD		RSCC		RSR		ΔE_{QM1}
				FeV	XO3	FeV	XO3	FeV	XO3	
CO_3	0	1	7.5	99.9	2.2	0.979	0.990	0.054	0.031	133
	0.01	1	7.5	99.9	1.6	0.981	0.989	0.050	0.033	110
	0.03	1	7.5	21.9	1.1	0.994	0.991	0.028	0.031	123
	0.1	1	7.5	11.5	0.9	0.997	0.991	0.019	0.030	132
	0.3	1	7.5	4.6	1.1	0.999	0.992	0.013	0.028	147
	1	1	7.5	2.9	1.6	0.999	0.991	0.012	0.029	171
	3	1	7.5	2.6	1.1	0.999	0.991	0.012	0.029	199
	10	1	7.5	3.1	0.4	0.999	0.993	0.012	0.026	229
	30	1	7.5	2.9	0.3	0.999	0.993	0.012	0.027	357
	100	1	7.5	1.8	0.1	0.999	0.995	0.011	0.023	498
	auto	1	7.5	2.5	0.6	0.999	0.994	0.012	0.025	209
	1	0	-	3.0	0.0	0.999	0.995	0.012	0.021	2235
	auto	1	-	2.1	0.5	0.999	0.993	0.012	0.027	2515
	deposited			3.2	0.1	0.999	0.995	0.012	0.022	284
HCO_3	0	1	7.5	99.9	4.2	0.974	0.976	0.058	0.048	118
	0.01	1	7.5	99.9	2.8	0.979	0.979	0.053	0.046	122
	0.03	1	7.5	26.3	3.2	0.991	0.978	0.033	0.050	120
	0.1	1	7.5	11.3	2.3	0.998	0.984	0.017	0.042	138
	0.3	1	7.5	5.9	2.2	0.999	0.985	0.014	0.039	149
	1	1	7.5	2.1	1.6	0.999	0.990	0.011	0.030	179
	3	1	7.5	3.1	1.2	0.999	0.991	0.012	0.028	212
	10	1	7.5	2.9	0.4	0.999	0.993	0.012	0.026	260
	30	1	7.5	3.1	0.1	0.999	0.994	0.012	0.024	359
	100	1	7.5	2.6	0.2	0.999	0.994	0.012	0.024	560
	auto	1	7.5	3.0	1.1	0.999	0.990	0.011	0.031	220
	1	0	-	2.2	0.1	0.999	0.994	0.012	0.022	2206
	auto	1	-	2.0	0.6	0.999	0.991	0.012	0.030	2580
	deposited			3.2	1.1	0.999	0.993	0.012	0.025	308
NO_3	0	1	7.5	99.9	3.7	0.978	0.983	0.053	0.037	117
	0.01	1	7.5	99.9	3.6	0.977	0.984	0.054	0.037	110
	0.03	1	7.5	99.9	3.7	0.984	0.981	0.045	0.040	111
	0.1	1	7.5	11.5	2.8	0.998	0.985	0.018	0.037	136
	0.3	1	7.5	5.2	1.5	0.999	0.990	0.014	0.031	142
	1	1	7.5	2.2	1.2	0.999	0.992	0.012	0.028	174
	3	1	7.5	2.2	1.6	0.999	0.992	0.011	0.027	195
	10	1	7.5	2.3	1.1	0.999	0.993	0.011	0.026	246
	30	1	7.5	2.1	1.1	0.999	0.991	0.012	0.030	320
	100	1	7.5	2.3	1.1	0.999	0.990	0.011	0.031	514
	auto	1	7.5	2.5	1.6	0.999	0.989	0.012	0.034	193
	1	0	-	2.5	1.1	0.999	0.991	0.012	0.030	2185
	auto	1	-	2.5	1.1	0.999	0.992	0.012	0.028	2457
	deposited			3.2	4.0	0.999	0.984	0.012	0.036	286

Table S4. Results of normal Phenix real-space refinements of the Cu_D site of pMMO (i.e. without any QM calculations) at different values of the w_x weight factor (keeping $w_c = 1$). The table shows the Cu–ligand distances (in Å) for OD1 of Asn-227C, for NE2 of His-231C, NE2 of His-245C, O of HOH-406C (W1) and O of HOH-415C (W2). It also shows RSCC of the ten residues allowed to move in the refinement (corresponding to those in the largest QM system in the quantum-refinement calculations), as well as the average of these values (Av).

w_x	Cu–X distances (Å)					RSCC										
	N227	H231	H245	W1	W2	N227	H231	H245	Cu	W406	W415	D156	R165	H173	F177	Av
0.00	2.08	2.22	2.42	2.39	3.02	0.89	0.88	0.91	0.80	0.63	0.74	0.83	0.82	0.81	0.87	0.818
0.01	2.08	2.21	2.37	2.44	3.02	0.89	0.88	0.91	0.80	0.64	0.74	0.83	0.82	0.81	0.87	0.819
0.03	2.08	2.25	2.32	2.45	2.96	0.89	0.88	0.91	0.81	0.64	0.74	0.83	0.82	0.81	0.87	0.820
0.1	2.08	2.22	2.35	2.45	3.00	0.89	0.88	0.91	0.81	0.64	0.74	0.83	0.82	0.81	0.88	0.821
0.3	2.06	2.16	2.13	2.63	3.02	0.89	0.88	0.91	0.83	0.66	0.75	0.83	0.82	0.81	0.88	0.827
1	2.03	2.12	2.10	2.77	3.09	0.90	0.88	0.91	0.84	0.68	0.76	0.84	0.83	0.82	0.89	0.834
3	2.02	2.07	1.98	3.08	3.19	0.90	0.88	0.91	0.85	0.70	0.76	0.84	0.84	0.84	0.89	0.842
10	2.05	2.04	1.89	3.40	3.27	0.90	0.88	0.91	0.86	0.72	0.76	0.85	0.84	0.86	0.90	0.850
30	2.17	2.02	1.83	3.63	3.31	0.90	0.88	0.91	0.87	0.74	0.76	0.86	0.85	0.88	0.91	0.856
100	2.37	2.05	1.83	3.77	3.34	0.90	0.89	0.92	0.87	0.74	0.76	0.88	0.85	0.89	0.91	0.861
1000	2.96	2.25	2.24	3.82	3.34	0.90	0.88	0.93	0.86	0.70	0.74	0.88	0.86	0.90	0.90	0.856
10000	3.23	2.71	2.83	3.82	3.34	0.80	0.83	0.88	0.80	0.56	0.65	0.78	0.78	0.85	0.84	0.778
auto=4.4	2.01	2.07	1.95	3.16	3.21	0.90	0.88	0.91	0.86	0.71	0.76	0.85	0.84	0.85	0.90	0.844
deposited	2.21	2.00	1.50	3.81	3.30	0.90	0.89	0.92	0.86	0.71	0.67	0.85	0.83	0.86	0.89	0.840

Figure S1. Nuclear scattering-length $mF_o - DF_c$ difference-density maps of the (a) deposited and (b) quantum-refined (with $w_x = 1$) neutron structures of reduced MnSOD (7KKW). Negative densities are shown in red (–2.5σ; no positive densities are seen in this region at that level and no difference densities are seen at –3σ).

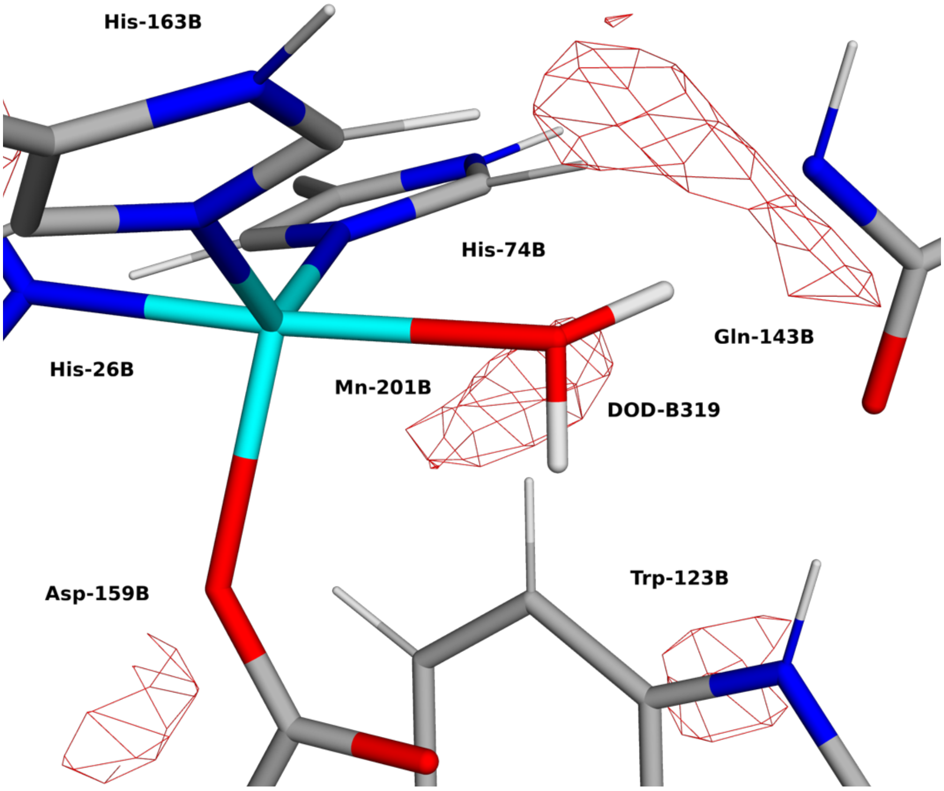

a

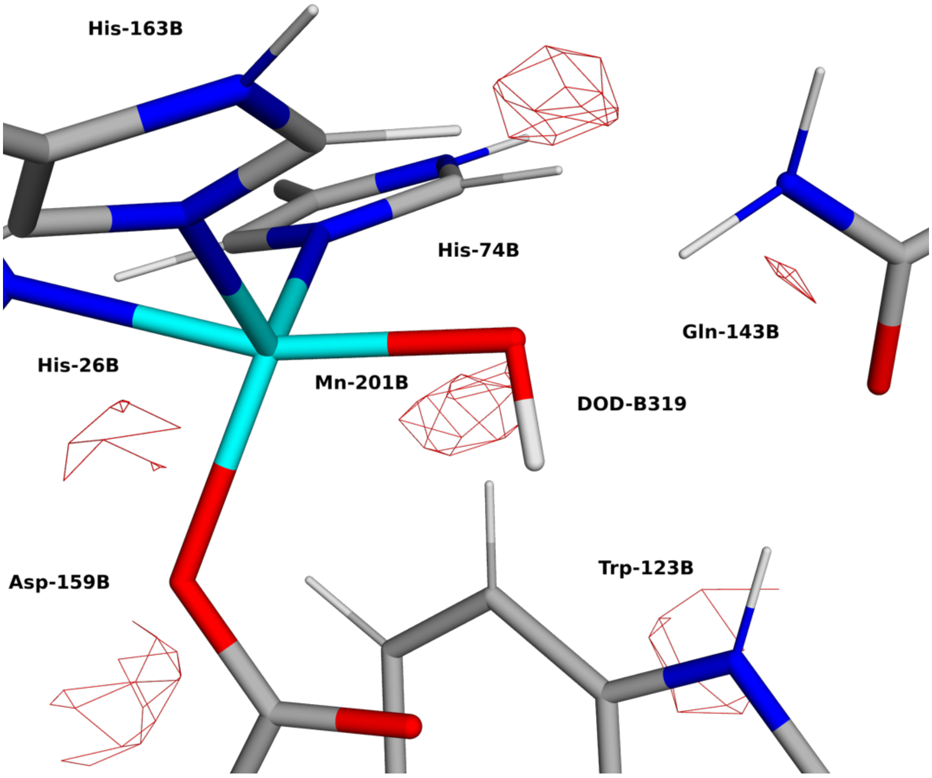

b

Figure S2. Relation between w_x, the strain energies (ΔE) and average RSZD for all ten residues in the QM system for the QRef calculations of V-nitrogenase with either CO_3^{2-}, HCO_3^- or NO_3^-.

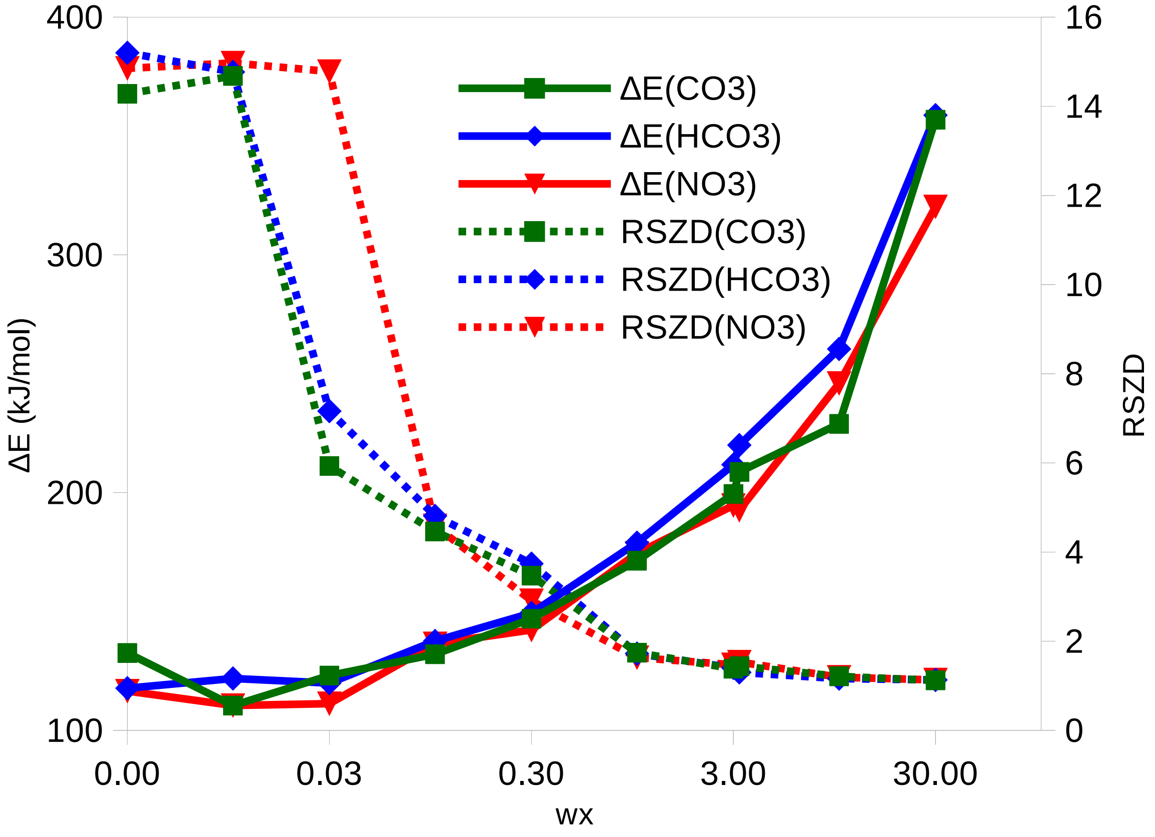

Figure S3. The $mF_o - DF_c$ difference-density maps of the V-nitrogenase with the bidentate ligand modelled by (a) CO_3^{2-}, (b) HCO_3^- or (c) NO_3^-, based on the quantum-refined structures with $w_x = 3$. Positive densities are shown in green (2.5σ) and negative densities in red (–2.5σ).

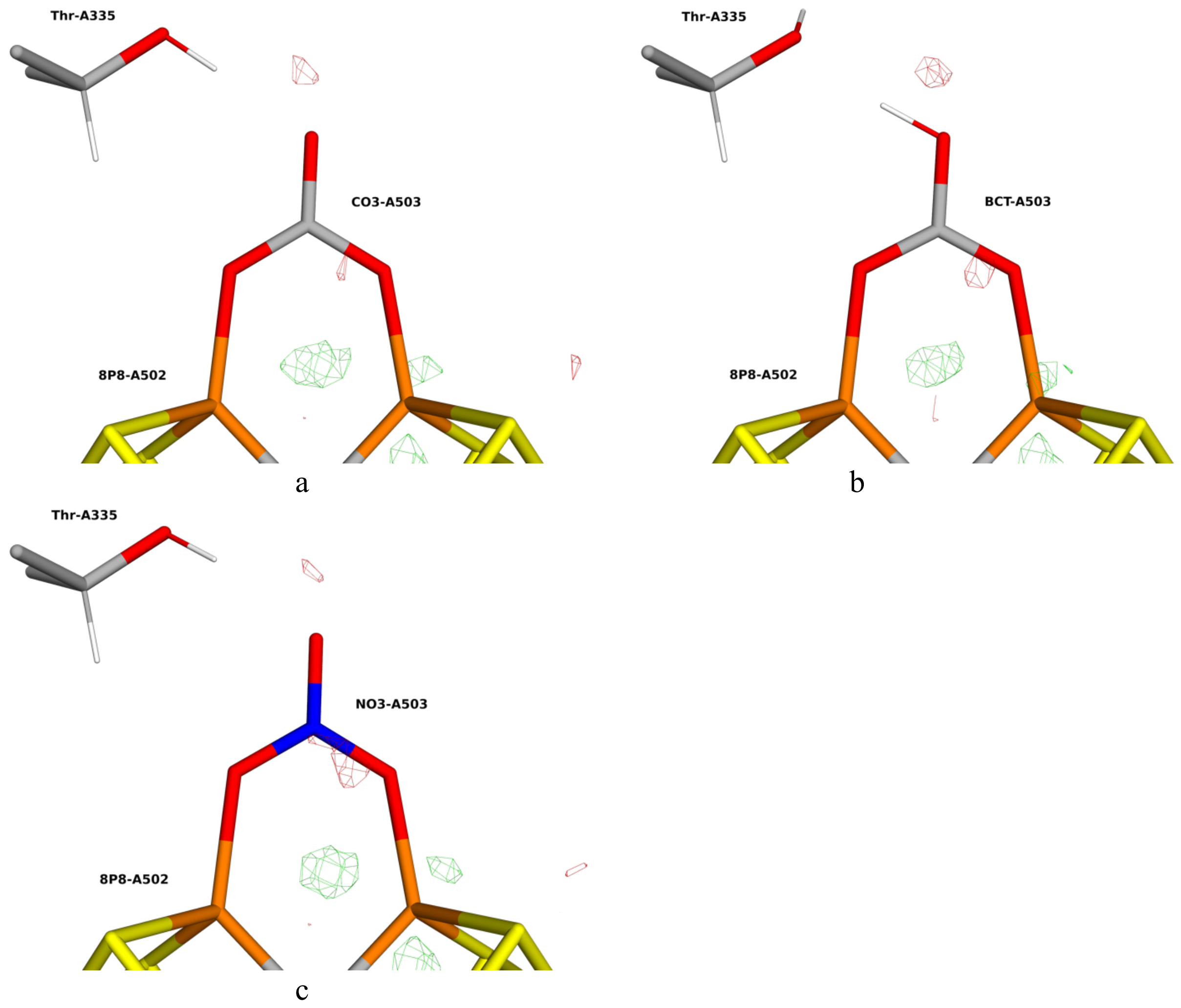

Figure S4. Relation between w_x, the strain energies (ΔE in kJ/mol) and average RSZD (a,b) or RSCC (c) scores for all ten residues in the QM system for the QRef calculations of (a) reduced MnSOD (subunit B), (b) Fe-nitrogenase and (c) the Cu_D site in pMMO, using the big QM region. The recommended w_x weight factors are 3, 0.9 and 3, respectively.

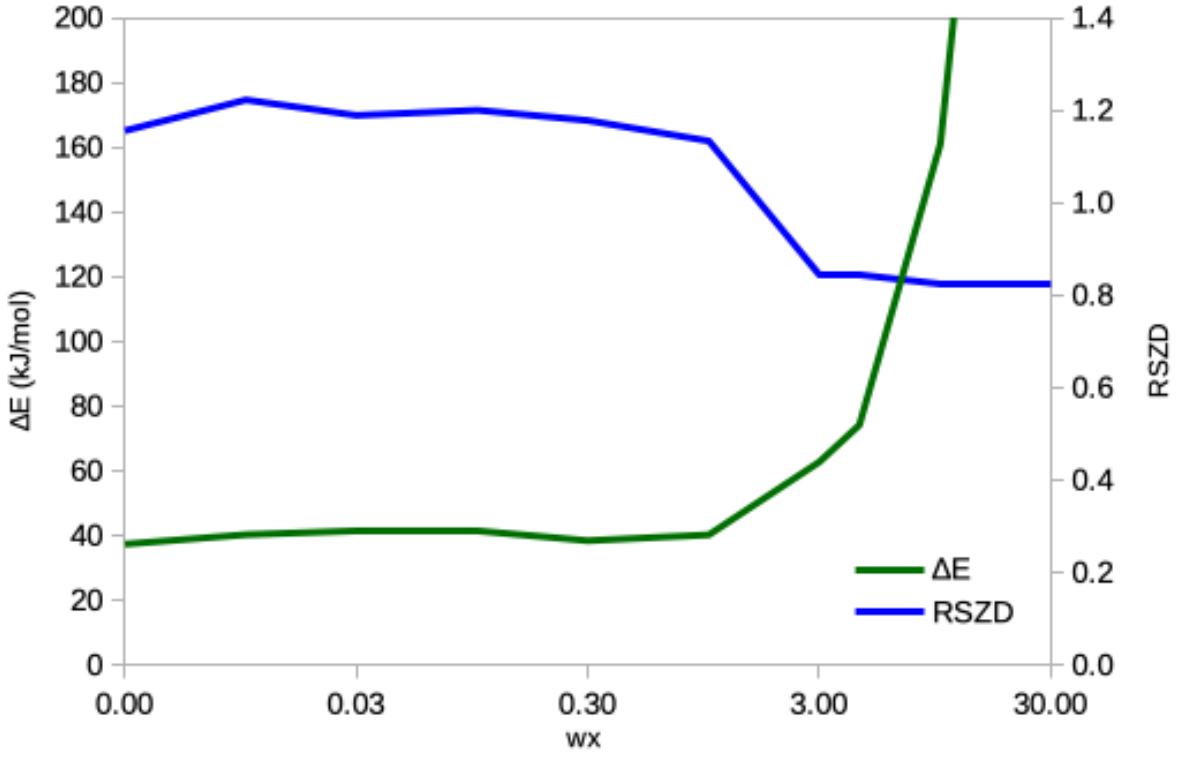

a

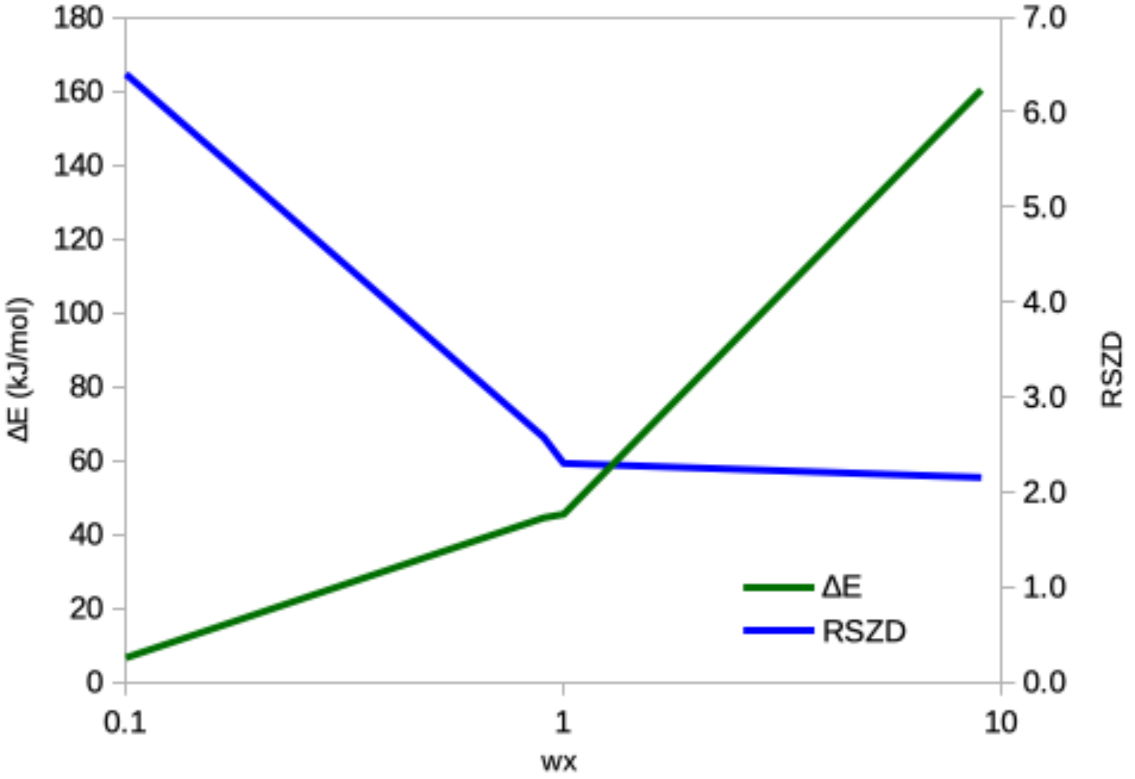

b

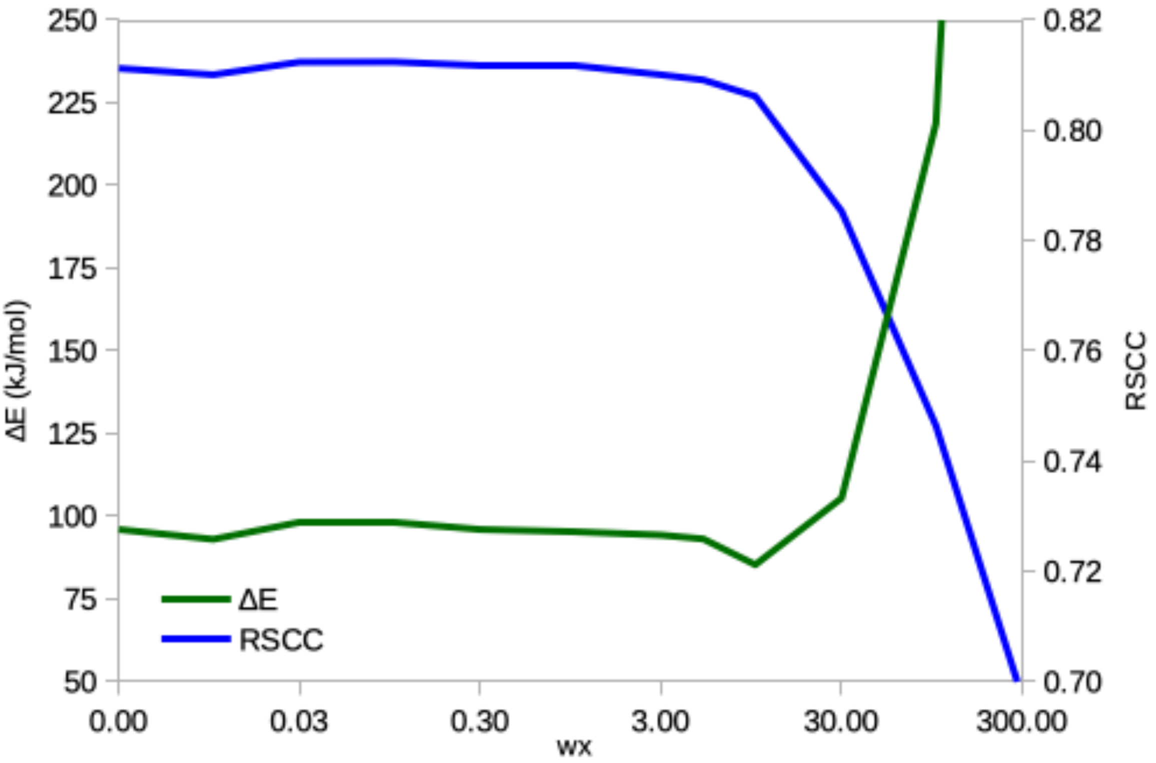

250
225
200
175
150
125
100
75
50
ΔE (kJ/mol)
0.82
0.80
0.78
0.76
0.74
0.72
0.70
RSCC
ΔE
RSCC
0.00
0.03
0.30
3.00
30.00
300.00
wx

c
